# Effects of Combinatorial Ubiquitinated Protein-Based Nanovaccine and STING Agonist in Mice With Drug-Resistant and Metastatic Breast Cancer

**DOI:** 10.3389/fimmu.2021.707298

**Published:** 2021-09-13

**Authors:** Fang Huang, Ning Pan, Yiting Wei, Jinjin Zhao, Mohanad Aldarouish, Xuru Wang, Xiaotong Sun, Zhifa Wen, Yongqiang Chen, Lixin Wang

**Affiliations:** Department of Microbiology and Immunology, Medical School of Southeast University, Nanjing, China

**Keywords:** ubiquitinated proteins, breast cancer, cancer vaccines, STING agonist, α-Al_2_O_3_ nanoparticles

## Abstract

We previously reported that enriched ubiquitinated proteins (UPs) from tumor cells have the potential to be used as immunotherapy vaccine against cancer. Here we enriched UPs from epirubicin (EPB)-induced multi-drug-resistant cancer stem-like breast cancer cell line (4T1/EPB) and tested the efficacy of α-Al_2_O_3_-UPs-4T1/EPB (short for UPs-4T1/EPB) as therapeutic vaccine alone and in combination with the stimulator of interferon genes (STING) agonist in mice with drug-resistant and metastatic breast cancer. Vaccination with UPs-4T1/EPB exerted profound anti-tumor effects through augmented specific CD8^+^ T cell responses and amplified T cell receptor diversity of tumor-infiltrating lymphocytes (TILs). Importantly, the combination with STING agonist further facilitated the migration of mature CD8α^+^ dendritic cells to the lymph nodes and the infiltration of TILs within tumors, resulting in primary tumor regression and pulmonary metastasis eradication in mice. Moreover, the cured mice were completely resistant against a subsequent rechallenge with the same tumor. Our study indicates that this novel combinatorial immunotherapy with UPs-4T1/EPB vaccine and STING agonist is effective in mice with drug-resistant and metastatic breast cancer.

## Introduction

Breast cancer is the most common malignancy and the second cause of cancer-related deaths in women worldwide (1). Chemotherapy remains the mainstay treatment option for breast cancer now (2). However, the therapeutic efficacy is often limited by multi-drug resistance (MDR) in that tumor cells develop resistance to one or more chemotherapy drugs, clinically presenting as a failure of chemotherapy, tumor metastasis, or tumor recurrence (3). Continuous efforts are made to develop novel therapeutic strategies for drug-resistant and metastatic breast cancer.

Immunotherapy is rapidly emerging as a promising therapeutic strategy against cancer (4). One of the most attractive immunotherapeutic strategies is therapeutic cancer vaccination, which is capable of eliminating the primary and metastatic tumor lesions through inducing the generation of antigen-specific cytotoxic T lymphocytes (CTLs) and specific immunological memory. Several cancer vaccines are currently under investigation for breast cancer, including whole-cell vaccines, peptide-based vaccines, gene-based vaccines, and dendritic cell (DC) vaccines (5). However, cancer vaccines have not yet achieved the expected clinical benefit in breast cancer patients mainly due to the failure to obtain sufficient tumor-associated antigens (TAAs) and initiate an efficient antigen presentation of TAAs (5, 6).

The efficient cross-presentation of TAAs by professional antigen-presenting cells (pAPCs) is the key to the success of cancer vaccines (6). In the effector phase of anti-tumor immune response, the peptides are directly presented by MHC-I molecules to the tumor cell surface and recognized by T cell receptors (TCRs) on antigen-specific CTLs (7). It is generally believed that most of these directly presented peptides are from short-lived proteins (SLiPs) with a half-life of around 10 min, which could be quickly ubiquitinated and then degraded by the ubiquitin–proteasome pathway (8). Thus, ubiquitinated proteins (UPs) could be viewed as SLiPs. However, the vast majority of the peptides cross-presented by pAPC to activate naive CD8^+^ T cells are from long-life proteins (LLiPs) with a half-life of around 3,000 min (8, 9). The discrepancy between the peptides cross-presented by pAPC (LLiPs), which are associated with naive T cell activation, and the peptides directly presented by MHC-I molecules to the tumor cell surface (SLiPs), which trigger the effector T cell attack, is considered as one of the major reasons for the limited spontaneous anti-tumor immunity by the tumor itself and the reason for the limited anti-tumor efficacy of several cancer vaccines (8, 9). Therefore, it is highly desirable to develop a cancer vaccine preparation strategy capable of enriching multiple SLiPs (UPs) in tumor cells and enhancing the cross-presentation of TAAs by pAPCs.

In previous studies, we demonstrated that the defective ribosomal products (DRiPs) containing blebs (Dribbles), which were enriched from tumor cells after the inhibition of proteosomal/lysosomal activity and the induction of autophagy, contained substantial amounts of SLiPs, and the anti-tumor efficacy of Dribbles vaccine was evidenced in various murine tumor models (10, 11). Further experiments identified that UPs were the major and efficient tumor antigen sources of DRibble vaccine (12). Recently, we showed that a ubiquitin binding protein (Vx3) covalently linked to α-Al_2_O_3_ nanoparticles could be used as a simple and effective platform to enrich UPs for the development of therapeutic cancer nanovaccines (13). There is growing evidence indicating that drug resistance- and cancer stem cell (CSC)-associated proteins could be the most promising targets for the design of a breast cancer vaccine (14, 15).

The cyclic GMP-AMP synthase (cGAS) stimulator of interferon genes (STING) pathway is a cytosolic DNA sensor in the endoplasmic reticulum (16). A STING agonist could lead to the activation of DCs, effective cross-priming of CD8^+^ T cells against tumor antigens, and migration of tumor-specific CD8^+^ T cells into the tumor (17). Recent studies found that STING agonists could significantly inhibit tumor growth in various mice models (18).

In this study, we established a novel epirubicin (EPB)-induced multi-drug-resistant CSC-like breast cancer cell line (4T1/EPB) and tested the hypothesis that STING agonist might enhance the anti-tumor efficacy of α-Al_2_O_3_-UPs-4T1/EPB (UPs-4T1/EPB for short) nanovaccine. A huge amount of drug resistance- and CSC-associated proteins was identified in 4T1/EPB cells, but not in wild-type 4T1 cells (4T1/WT), by mass spectrometry. More importantly, we demonstrated that vaccination with UPs derived from 4T1/EPB cells, but not from 4T1/WT cells, exerted profound anti-tumor effects through augmented cancer-specific CD8^+^ T cell responses and expanded the diversity of the TCR repertoire. UPs-4T1/EPB nanovaccine in combination with STING agonist (5,6-dimethylxanthenone-4-acetic acid; DMXAA) resulted in complete tumor regression and complete elimination of the metastases in the majority of mice with drug-resistant and metastatic breast cancer.

## Materials and Methods

### Mice

Six- to 8-week-old wild-type female BALB/c mice were purchased from QingLongShan Animal Center (Nanjing, China) and maintained in specific pathogen-free conditions. All animal experiments were approved by the Institutional Animal Care and Welfare Committee of Southeast University and strictly followed the animal welfare guidelines of the Institutional Animal Care and Welfare Committee of Southeast University.

### EPB-Resistant 4T1 Cell Line—Establishment and Identification

Until now there are no EPB-resistant metastatic mouse breast cancer cells. According to a previous report on inducing *in vitro* drug resistance (19), highly metastatic mouse breast cancer cells (4T1/WT cells) were exposed to increasing concentrations of EPB (Pfizer Pharmaceuticals, H19990280, USA), starting at half-maximum inhibitory concentration values (IC50; 3 µg/ml) and gradually increasing to 200 µg/ml over a period of 10 months. The IC50 of the cell line was determined by CCK8 toxicity test (Cell Counting Kit-8, Japan), and the resistance index (RI) was calculated as RI = IC50 of the drug-resistant cell line/IC50 of the parental generation cell line. Four repeats were tested (19). The drug resistance index of the cells was 20.5, and the newly established stable EPB-resistant 4T1 cell line was named 4T1/EPB.

To observe the *in vivo* drug sensitivity, 5 × 10^5^ 4T1/WT cells and 4T1/EPB cells were then challenged subcutaneously (s.c.) into the opposite flanks of 6–8-week-old BALB/c female mice, respectively. The mice received an intravenous (i.v.) injection of EPB (200 µg/mouse) or normal saline (NS) in a total volume of 200 µl on day 5 and day 10 (*n* = 6 per group). All mice survived up to the designed study endpoint and were sacrificed on day 30 under deep anesthesia by an intraperitoneal (i.p.) injection of 150 mg/kg pentobarbital sodium. The tumor tissues were dissected, the tumor was photographed, and the weight of the tumors was measured.

In separate experiments, different numbers (2 × 10^2^, 2 × 10^3^, 2 × 10^4^, 2 × 10^5^, and 2 × 10^6^) of 4T1/WT and 4T1/EPB cells were challenged s.c. into the opposite flanks of mice (*n* = 5 per group). The mice were examined every 3 days to observe the tumorigenicity of these two cells in various concentrations. All mice survived up to the designed study endpoint and were sacrificed on day 20 under deep anesthesia (pentobarbital sodium, 150 mg/kg, i.p.). The flanks of the mice were shaved with an electric razor, and the mice were photographed.

### Quantitative Real-Time PCR

Total RNA was extracted from tumor cells using TRIzol following the standard isolation protocol (Invitrogen, USA) followed by reverse transcription into cDNA with Prime-Script RT reagent Kit (Takara, China). Quantitative real-time PCR was performed using Fast Start Universal SYBR Green Master (ROX) (Roche Life Science, USA) on StepOne Real-Time PCR System (Thermo Fisher Scientific, USA). The primer sequences (Sangon Biotech Shanghai, China) were as follows: multi-drug resistance protein 1 (MDR1)-F: ATCATCAGCAACAGCAGTCTGGA, MDR1-R: GGCACCAGTGAAACCTGGA; breast cancer resistance protein (BCRP)-F: ACGACTGGTTTGGACTCAAGCAC, BCRP-R: AAAGATGGAATACCGAGGCTGATG; glutathione S transferase- π (GST-π)-F: CTCTGTCTACGCAGCACTGAA-TC, GST-π-R: CAAGCCTTGCATCCAGGTATC; and matrix metallopeptidase 7 (MMP7)-F: CAGACTTACCTCGGATCGTA-GTGGA, MMP7-R: TGCGAAGGCATGACCTAGAGTG.

### Western Blotting

The tumor cells were lysed in RIPA lysis buffer containing protease inhibitors (MedChemExpress, USA) and phosphatase inhibitors (MedChemExpress, USA), and MDR1 was determined using anti-MDR1 (1:1,000, Abcam, ab170904, UK) as the primary antibody and goat anti-rabbit IgG HRP as the secondary antibody (1:5,000, eBioscience, USA). Actin served as the endogenous control.

For ubiquitin detection, an equal amount of protein (20 µg) was loaded from all samples, as assessed by Coomassie blue-stained SDS-PAGE. Anti-ubiquitin antibody (1:1,000, Sigma, #3933, USA) served as the primary antibody, and goat anti-rabbit IgG HRP (1:5,000, eBioscience, USA) served as the secondary antibody.

### Flow Cytometry and Antibodies

A single-cell suspension was blocked with mouse FcR blocking reagent (Miltenyi Biotec, USA) at 4°C for 10 min prior to surface staining. For intracellular IFN-γ staining, the cells were fixed and permeabilized with Fixation/Permeabilization Kit (BD Biosciences, USA) and then stained with IFN-γ antibody. The following anti-mouse antibodies were used: PE-CD44 (clone:IM7), APC-CD24 (clone:M1-69), FITC-CD3e (clone: 145-2C11), PE-CD45 (clone:30-F11), APC-MHC class II (I-A/I-E) (clone: AMS-32.1), PE-CD86 (clone: GL 1) from eBioscience (San Diego, CA, USA); PE/Cy7-CD8a (clone: 53-6.7), PE-MHC class I (H-2Kd) (clone: AMS-32.1), PE/Cy7-CD40 (clone: 3-23), PE/Cy7-CD11c (clone: HL3) from BD Biosciences (USA); and APC-CD8a (clone: 53-6.7), PE-IFN-γ (clone: XMG 1.2), APC-CD11c (clone:N418), APC-CD80 (clone: 16-10A1) from Biolegend (USA). For ALDH1 staining, the ALDH1 activity was detected by the ALDEFLUOR kit (Stem Cell Technologies, Canada) according to the instructions of the manufacturer. Data were acquired on BD FACS Calibur (USA) and analyzed by the Flow-Jo software (Tree Star, OR) version 7.1.2. Fixable viability dye eFluor 520 (eBioscience, USA) was used to exclude dead cells.

### *In Vitro* Cell Migration and Invasion Assays

For the migration assay, 100 µl serum-free medium containing 2 × 10^5^ cells was added to the upper chambers of 24-well Transwell chambers (8-mm pore size; Corning, USA). For the invasion assay, 4 × 10^5^ cells in 100 µl serum-free medium were plated into the upper chambers precoated with Matrigel (Corning, USA). The lower chamber was filled with 600 µl complete culture medium. Then, the cells were cultured in an incubator at 37°C under 5% CO_2_ and saturated humidity condition for 24 h. The cells in the lower chamber were fixed in 4% paraformaldehyde (Servicebio Technology, China) for 30 min, stained with 0.1% crystal violet for 20 min, and then counted under a microscope according to the protocol of the manufacturer.

### Preparation of UP Nanovaccine

Vx3 protein was obtained and covalently linked to α-Al_2_O_3_ nanoparticles to generate α-Al_2_O_3_-Vx3 nanoparticles according to our previous reports (12, 13). 4T1/WT cells and 4T1/EPB cells were treated with 200 nM bortezomib (Millennium Pharmaceuticals, USA) and 20 mM NH_4_Cl (Sigma, USA) for 9 h. The cells were collected and lysed in RIPA lysis buffer (Millipore, USA) containing protease inhibitors (MedChemExpress, USA), phosphatase inhibitors (MedChemExpress, USA), and PR-619 (MedChemExpress, USA). The cell lysate was reacted with α-Al_2_O_3_-Vx3 nanoparticles under stirring for 12 h at 4°C to generate α-Al_2_O_3_-UPs nanovaccine (named UP-nanovaccine) according to our previous study (13, 20). The precipitates (UP-nanovaccine) were collected by centrifugation (12,000 *g*, 30 min, 4°C), and the supernatant was collected as unbound lysate, followed by the detection of ubiquitin protein levels in the three samples using Western blotting. The covalently linked product UP-nanovaccine was collected by centrifugation, and the number of UPs enriched by α-Al_2_O_3_-Vx3 was evaluated by collecting and calculating the difference between the number of UPs in the supernatant before and after the reaction by BCA Protein Assay Kit (Beyotime Biotechnology, China) according to the protocol of the manufacturer.

### UP Protein Identification by Label-Free LC–MS/MS Analysis

The UPs-4T1/WT nanovaccine and UPs-4T1/EPB nanovaccine obtained from 4T1/WT cells and 4T1/EPB cells underwent trypsinolysis after extraction and digestion. Sample peptides were separated by LC-20AD liquid chromatography (Shimadzu, Japan). The sample was loaded onto a self-packed C18 column and then separated by gradient steps. The peptides were ionized by a nanoESI source and then passed to a triple time-of-flight 5600 mass spectrometer (SCIEX, USA). The mass spectrometry data were processed using MaxQuant (version 1.5.3.30) (21), and its built-in search engine Andromeda was used to perform database searches against Mouse entries in the UniprotKB/SwissProt protein database for peptide identification and protein inference. Mass spectrometry and protein identification services were provided by the HuaDa Gene Company (China). The mass spectrometry proteomics data have been deposited to the ProteomeXchange Consortium (http://www.ebi.ac.uk/pride) *via* the PRIDE (22) partner repository, with the dataset identifier PXD027822. Venn diagrams were generated using BioVenn (23).

### Anti-Tumor Effect of UPs-4T1/WT Nanovaccine and UPs-4T1/EPB Nanovaccine

To assess the prophylactic anti-tumor effects of UP-nanovaccine, the mice received a s.c. injection of NS, UPs-4T1/WT nanovaccine (containing 30 µg UPs), or UPs-4T1/EPB nanovaccine (containing 30 µg UPs), in a total volume of 200 µl, three times at 2-day intervals (12 mice per group) (13, 20). The vaccines were injected s.c. on two sides of the axilla of the mice as previously described (13, 20). At 7 days after the last immunization, five mice from each group were sacrificed under deep anesthesia, and the splenocytes were separated. The splenocytes were then restimulated with inactivated 4T1/WT or 4T1/EPB cells treated with Mitomycin C (Sigma, USA) for 24 h. The splenocytes stimulated with anti-CD3 Ab only were used as the positive control, while culture medium was used as the negative control. The IFN-γ^+^ CD3^+^ CD8^+^ T cells were examined by flow cytometry. The supernatants were collected and examined for IFN-γ with an ELISA assay. The remaining mice (seven mice per group) received a tumor challenge s.c. with 5 × 10^5^ 4T1/WT or 4T1/EPB cells 2 days after the last immunization. The tumor cells were s.c. injected into the right second mammary fat pad of each mouse as previously described (13). The tumor size was measured every 3 days with a Vernier caliper using the following formula: tumor volume (mm^3^) = (length × width^2^)/2. The mice were humanely sacrificed when the tumor reached 2,000 mm^3^. The surviving mice were sacrificed under deep anesthesia on day 50; the liver, lung, spleen, and peritoneum were isolated and examined for metastases. The metastases rate was expressed by dividing the number of mice with metastases by the total number of mice.

To assess the therapeutic anti-tumor effects of UP-nanovaccine, the mice were challenged s.c. with 5 × 10^5^ 4T1/WT cells or 4T1/EPB cells on day 0 and received s.c. immunization with NS, UPs-4T1/WT nanovaccine (containing 30 µg UPs), or UPs-4T1/EPB nanovaccine (containing 30 µg UPs; 12 mice per group), in a total volume of 200 µl, on days 8, 10, and 12. On day 15, six mice from each group in the 4T1/EPB tumor-bearing mice were sacrificed under deep anesthesia, and the tumor tissues were separated for CD8^+^ TILs isolation. The isolated CD8^+^ TILs from each group were mixed together as a pooled sample, and the TCRs of CD8^+^ TILs from each sample were profiled with high-throughput TCR sequencing. The TCRs of CD8^+^ TILs were profiled with high-throughput TCR sequencing. The remaining mice (*n* = 6 each group) were used for observations of tumor growth and survival until day 50. Then, the surviving mice were sacrificed under deep anesthesia; the liver, lung, spleen, and peritoneum were isolated and examined for metastases.

To evaluate the combination therapy of UPs-4T1/EPB nanovaccine and chemotherapy in the 4T1/EPB tumor-bearing study, the mice were challenged s.c. with 5 × 10^5^ 4T1/EPB cells on day 0 and received an i.v. injection of 0 or 100 µg EPB, in a total volume of 200 µl, on day 5 (20, 24), followed by a s.c. injection of NS or UPs-4T1/EPB nanovaccine (containing 30 µg UPs), in a total volume of 200 µl, three times on days 8, 10, and 12 (five mice per group). The tumor growth and survival of mice were monitored until day 50. Then surviving mice were sacrificed under deep anesthesia; the liver, lung, spleen, and peritoneum were isolated and examined for metastases.

To evaluate the effect of UPs-4T1/EPB nanovaccine combined with DMXAA in the 4T1/EPB tumor-bearing study, the mice were inoculated with 5 × 10^5^ 4T1/EPB cells s.c. on day 0 and received an i.v. injection of 100 µg EPB, in a total volume of 200 µl, on day 5. The mice then received a s.c. vaccination of NS, UPs-4T1/WT nanovaccine (containing 30 µg UPs), and UPs-4T1/EPB nanovaccine (containing 30 µg UPs) alone or in combination with 100 µg DMXAA (MedChemExpress, #117570-53-3, USA), in a total volume of 200 µl, three times on days 8, 10 and 12. The tumor growth and survival of mice were monitored until day 50. Then, the surviving mice were sacrificed under deep anesthesia; the liver, lung, spleen, and peritoneum were isolated and examined for metastases. Lung metastasis was selected to further evaluate the tumor metastasis in the subsequent experiments for the following reasons: first, lung metastasis is easier and more accurate to observe, and it is an easy way to calculate the metastatic nodules involved with a clear boundary; second, the lung was the preferential and major involved metastatic organ of this model (25, 26). Therefore, the lungs were fixed in Bouin’s solution followed by hematoxylin and eosin (HE) staining, and the lung metastatic nodules were counted.

To perform the tumor rechallenge assays in UPs-4T1/EPB nanovaccine combined with chemotherapy and DMXAA in cured mice 50 days after tumor inoculation, the cured mice were used as the immunization group and the untreated BALB/c mice were used as the control group (five mice per group). The mice were subsequently injected with 5 × 10^5^ 4T1/EPB cells. The tumor growth was measured util 45 days after the tumor rechallenge.

To detect the anti-tumor effects of UPs-4T1/EPB nanovaccine combined with DMXAA in CD4^+^ T cell-depleted or CD8^+^ T cell-depleted mice, the mice were injected with 5 × 10^5^ 4T1/EPB cells s.c. on day 0 and received an i.v. injection of 100 µg EPB, in a total volume of 200 µl, on day 5 (four mice per group). The mice then received a s.c. vaccination of NS or UPs-4T1/EPB nanovaccine (containing 30 µg UPs) combined with 100 µg DMXAA three times, in a total volume of 200 µl, on days 8, 10, and 12. CD4^+^ or CD8^+^ T cell depletion was performed *via* an i.p. injection of CD4 (250 µg/200 µl/mice, BioXCell, clone GK1.5, USA) or CD8 antibodies (250 µg/200 µl/mice, BioXCell, clone 2.43, USA). At 1 day before the combination therapy, the mice received an i.p. injection of depletion antibodies every 4 days throughout the course of tumor growth. Tumor growth was monitored until day 30, and all mice were euthanized under deep anesthesia on day 30. The liver, lung, spleen, and peritoneum were isolated and examined for metastases. The tumor weight and lung metastatic nodules of Bouin’s-fixed lungs were counted.

To evaluate the combination therapy of UPs-4T1/EPB nanovaccine and DMXAA in mice with a larger breast tumor, the mice were challenged s.c. with 5 × 10^5^ 4T1/EPB cells on day 0 and received an i.v. injection of EPB (100 µg/mouse), in a total volume of 200 µl, on day 5. The vaccine treatment was postponed, and the mice received a s.c. injection of UPs-4T1/EPB nanovaccine (containing 30 µg UPs) with or without a combination of 100 µg DMXAA, in a total volume of 200 µl, on day 14 for three times at 2-day intervals (four mice per group). Tumor growth was monitored until day 50, and the surviving mice were euthanized under deep anesthesia. The liver, lung, spleen, and peritoneum were isolated and examined for metastases. The metastatic nodules of Bouin’s-fixed lungs were counted.

To further detect the effect of combination therapy with UPs-4T1/EPB vaccine and αPD-L1 treatment in 4T1/EPB tumor-bearing mice, the mice were challenged s.c. with 5 × 10^5^ 4T1/EPB cells on day 0 and received an i.v. injection of EPB (100 µg/mouse), in a total volume of 200 µl, on day 5, followed by a s.c. injection of NS or UPs-4T1/EPB nanovaccine (containing 30 µg UPs) three times on days 8, 10, and 12, and then they received an i.p. injection of 0 or 200 µg αPD-L1 (clone MIH5, BioXCell, USA), in a total volume of 200 µl, on days 15 and 20 (six mice per group). The tumor growth and survival of mice were monitored until day 50. Then, the surviving mice were sacrificed under deep anesthesia; the liver, lung, spleen, and peritoneum were isolated and examined for metastases. The lungs were separated and fixed in Bouin’s solution, followed by H&E staining, and the lung metastatic nodules were counted.

### Immune Response Detection

The mice were challenged s.c. with 5 × 10^5^ 4T1/EPB cells on day 0. The mice then received an i.v. injection of EPB (100 µg/mouse), in a total volume of 200 µl, on day 5 and received a s.c. injection of UPs-4T1/EPB nanovaccine (containing 30 µg UPs) alone or combined with 100 µg DMXAA, in a total volume of 200 µl, on days 14, 16, and 18 three times at 2-day intervals (five mice per group). The mice were euthanized 3 days after the last immunization under deep anesthesia, and the DLNs, spleen, and tumor tissues were dissected and separated for subsequent experiments. The spleen cells of mice were harvested. The percentage of CD8^+^ T cells, CD4^+^ T cells, and total T cells in spleens was analyzed by flow cytometry, and the absolute numbers were calculated. The splenocytes were stimulated with inactivated 4T1/EPB cells for 24 h. The percentage of IFN-γ^+^ CD3^+^ CD8^+^ T cells was evaluated by flow cytometry, and the absolute numbers were calculated. The total IFN-γ level (Invitrogen, #88-7314-77CA, USA)in the cell supernatant was detected by ELISA.

The DLNs of mice were harvested. The percentage of total DCs and CD8α^+^ DCs was analyzed by flow cytometry, and the absolute numbers were calculated. The expression of CD80, CD86, and MHC class I and II on CD8α^+^ DCs was examined by flow cytometry.

The tumor tissues of mice were harvested. Half of each tumor was isolated and processed to a single-cell suspension, and the percentage of total T lymphocytes (CD45^+^ CD3^+^ cells) and T cell subsets (CD45^+^ CD3^+^ CD8^+^ T cells and CD45^+^ CD3^+^ CD8^-^ T cells) was detected using flow cytometry. The other half of tumor tissue was immunofluorescent-stained with PE-CD8 antibody, FITC-ki67 antibody, and DAPI. The Ki-67^+^ CD8^+^ cells were observed and counted with a confocal microscope.

### High-Throughput TCR Sequencing

The CD8^+^ TILs in tumor tissues were separated and purified by CD8 (TIL) magnetic microbeads kit (Miltenyi Biotec, #130-116-478, USA) at 2 days after the last immunization, and the RNA from CD8^+^ TILs was extracted using Trizol, followed by reverse transcription to DNA for high-throughput TCR sequencing. Sample data were generated using the immune-SEQ assay. TCR α, TCR β, and their complementarity determining regions (CDRs) 1–3 were amplified and sequenced using the I Illumina Hiseq (27). For each sequence, V and J genes and alleles, CDRs 1–3 length, and nucleotide sequences were identified using the IMGT database. High‐throughput TCR sequencing services were provided by GENEWIZ Company (China).

### Detection of DC Activation and Antigen Presentation

Bone marrow-derived DCs (BMDCs) were prepared according to the previous literatures (20) and co-cultured with 10 µg/ml DMXAA, 10 µg/ml UPs-4T1/EPB nanovaccine, and UPs-4T1/EPB nanovaccine combined with DMXAA for 24 h. The expression of BMDC surface activation molecules (MHC-I, MHC-II, CD40, CD80, and CD86) was detected by flow cytometry. The level of IFN-β and IL-12p70 (Biolegend, #439407, USA; Invitrogen, #88-7121-22, USA) in the culture supernatant was detected by ELISA. UPs-4T1/EPB nanovaccine alone or combined with DMXAA was incubated with splenocytes from UPs-4T1/EPB nanovaccine immunized mice for 12 h and the IFN-γ in the culture supernatant was detected by ELISA.

### Evaluation on Safety and Toxicity of UP Nanovaccine in Mice

The mice received a s.c. injection of NS, UPs-4T1/WT nanovaccine (containing 30 µg UPs), or UPs-4T1/EPB nanovaccine (containing 30 µg UPs), in a total volume of 200 µl, three times at 2-day intervals on day 6, 8, and 10 (six mice per group). No death occurred during this period, and the mice were euthanized 21 days after the last immunization. The liver, lung, and kidney were collected for HE staining to observe pathological tissues, and the ultrastructure of the kidney tissues was observed using a transmission electron microscope (TEM) (Hitachi, HT770, Japan).

### Statistical Analysis

Statistical analysis was performed using Graph-Pad Prism 7 for Windows (GraphPad Prism, San Diego, USA), and experimental data came from at least three independent experiments. All collected data were firstly tested for normal distribution by D’Agostino’s K-square test. The two-tailed unpaired Student’s *t*-test was applied to compare two normally distributed groups, and the Mann–Whitney U-test was applied to compare two groups for those which did not, respectively. When multiple groups were compared, one-way analysis of variance (one-way ANOVA) (28) was used for data that fulfilled normal distribution, and the Kruskal–Wallis test was used for those that did not, respectively. Kaplan–Meier survival curves were assessed by the log-rank Mantel–Cox test. Data were expressed as means ± SEM (*p < 0.05; **p < 0.01; ***p < 0.001; ****p < 0.0001; ns, not significant), and *P <*0.05 was considered significant.

## Results

### Enriched Drug Resistance- and Breast Cancer Stem Cell-Associated Proteins in UPs Derived From Newly Established 4T1/EPB Cells

**Figure 1:**
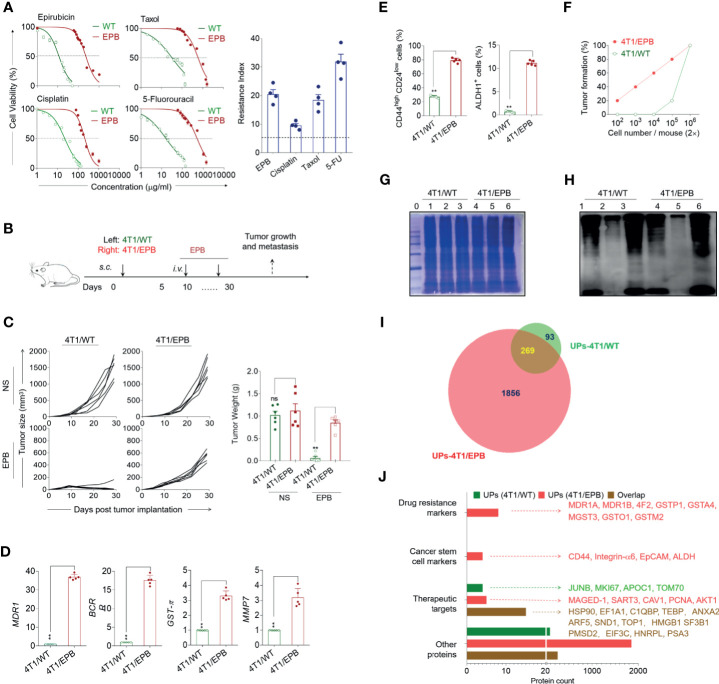
Multi-drug-resistant and breast cancer stem cell-like features of the newly established 4T1/epirubicin (EPB) cells and expanded ubiquitinated protein (UP) spectrum with related markers and therapeutic target-associated proteins. **(A)** The cell viability of 4T1/WT cells and 4T1/EPB cells was assessed at varying concentrations of EPB, cisplatin, Taxol, and 5-fluorouracil, respectively, to calculate IC50 values and resistance index. **(B)** Scheme of the *in vivo* drug sensitivity experimental protocol. **(C)** Tumor growth and tumor weight of tumor-bearing mice (*n* = 6 per group) in **(B)**. **(D)** qRT-PCR analysis was applied to the expression of MDR1, BCRP, GST-π, and MMP7 in 4T1/WT and 4T1/EPB cells. **(E)** The expression of CD44^high^CD24^low^ population and ALDH1 activity on 4T1/WT cells and 4T1/EPB cells was determined by flow cytometry. **(F)** The tumorigenicity of 4T1/WT cells and 4T1/EPB cells in BALB/c mice was detected (*n* = 5 per group). **(G)** The equal protein loading of the protein marker (lane 0), whole cell lysate (lane 1 and lane 4), unbound lysate (lane 2 and lane 5), and α-Al_2_O_3_-Vx3-UPs (lane 3 and lane 6) from 4T1/WT cells and 4T1/EPB cells was verified by SDS-PAGE electrophoresis and Coomassie blue stain. **(H)** The protein levels of the ubiquitin in **(G)** were determined by western blot. **(I)** Venn diagram showing the overlap between the 362 proteins identified in UPs from 4T1/WT cells by LC–MS/MS analysis (green) and the 2,125 proteins identified in UPs from 4T1/EPB cells (red). **(J)** Bar chart showing the number of proteins classified according to the following categories: drug resistance markers, cancer stem cell markers and therapeutic targets, and other proteins in **(I)**. *P*-values were determined by Mann–Whitney *U*-test. The results are representative of three independent experiments, and data were expressed as means ± SEM (**p < 0.01; ns, not significant).

We characterized the features of the newly established EPB-induced multi-drug-resistant CSC-like breast cancer cell line (4T1/EPB) and UPs derived from 4T1/EPB cells. It was shown that 4T1/EPB cells exhibited cross-resistance to cisplatin, Taxol and 5-fluorouracil (Drug resistance index: 9.6, 18.5, and 31.9, respectively; **Figure 1A**). The *in vivo* experiments showed that the s.c. inoculation of 4T1/EPB cells significantly reduced the chemosensitivity to EPB compared to the 4T1/WT inoculation (**Figures 1B, C** and **Supplementary Figure S1A**). Moreover, the qRT-PCR analysis evidenced significantly upregulated drug resistance-related genes, including MDR1, BCRP, GST-π, and MMP7 in 4T1/EPB cells compared to the 4T1/WT cells (fold increase of resistance: 36.9, 17.7, 3.3, and 3.2 times, respectively; **Figure 1D**). Consistently, the protein level of MDR1 was also significantly upregulated in 4T1/EPB cells than in 4T1/WT cells (**Supplementary Figure S1B**). These results indicate that the newly established 4T1/EPB cells display multi-drug-resistant characteristics.

It is known that CSCs are related to drug resistance (3). We thus defined the CSC properties of 4T1/EPB cells. The percentage of CD44^+^/CD24^−^ cell population (81 *vs*. 28%) and ALDH1 expression (11.6 *vs*. 0.8%) was significantly higher in 4T1/EPB cells than in 4T1/WT cells (**Figure 1E** and **Supplementary Figure S1C**). Furthermore, *in vivo* tumorigenicity assays showed that 2 × 10^2^ 4T1/EPB cells were sufficient to induce tumor formation in mice, whereas 2 × 10^5^ 4T1/WT cells were required to induce tumor formation in mice (**Figure 1F** and **Supplementary Figure S1D**). Concomitantly, the migration and invasion capabilities of 4T1/EPB cells were significantly stronger than those of 4T1/WT cells (**Supplementary Figures S1E, F**). Taken together, these results indicate that the established multi-drug-resistant breast cancer cells (4T1/EPB cells) display the characteristic features of BCSCs (29).

Next, the whole-cell lysates were collected from 4T1/WT cells and 4T1/EPB cells according to the established method; the UPs were then enriched by α-Al_2_O_3_-Vx3 nanoparticles (13). The whole-cell lysate, the UPs bound to the nanoparticles (α-Al_2_O_3_-Vx3-UPs), and the leftover lysate after nanoparticle binding (unbound lysate) were subject to ubiquitin detection by western blot with anti-ubiquitin antibody. The SDS-PAGE electrophoresis and Coomassie blue stain results ensured that an equal total protein amount was loaded from the three samples (**Figure 1G**), and the ubiquitin level was significantly higher in UPs than in the whole-cell lysate, and only a small amount of ubiquitin was detected in the unbound lysate (**Figure 1H**). These results confirmed the efficient enrichment of UPs from the whole-cell lysate of 4T1/WT and 4T1/EPB cells by the applied procedure.

The UPs from the 4T1/WT cells and the 4T1/EPB cells were obtained by α-Al_2_O_3_-Vx3 and then analyzed by mass spectrometry. The Venn diagram showed that a total of 362 proteins were identified in 4T1/WT-derived UPs, whereas 2,125 proteins were identified in 4T1/EPB-derived UPs, among which 269 proteins were overlapping (**Figure 1I** and **Supplementary Tables S1**, **S2**). Related PubMed literatures (http://www.ncbi.nlm.nih.gov/pubmed/) were searched to explore the potential association between identified proteins and breast cancer. Based on the search results, the proteins were classified into the following four groups: drug resistance markers, CSC markers, therapeutic targets (excluding the former two protein categories), and other proteins (**Figure 1J**). Notably, drug resistance- and CSC-associated proteins were identified exclusively in 4T1/EPB-derived UPs, including eight breast cancer-associated drug-resistant proteins: MDR1A, MDR1B, 4F2, GSTP1, GSTA4, MGST3, GSTO1, and GSTM2 (14); four breast cancer-associated stem cell proteins: CD44, integrin-α6, EpCAM, and ALDH1 (15, 29); and five therapeutic target-associated proteins: MAGED-1, SART3, CAV1, PCNA, and AKT1 (30, 31). Moreover, 14 therapeutic target-associated proteins, including HSP90, EF1A1, C1QBP, TEBP, ANXA2, ARF5, SND1, TOP1, HMGB1, SF3B1, PMSD2, EIF3C, HNRPL, and PSA3 (32, 33), were identified in both 4T1/WT- and 4T1/EPB-derived UPs. Moreover, gene ontology functional enrichment analysis of UPs was performed in **Supplementary Figure S1G**. Collectively, these results indicate that the UP spectrum is expanded in the 4T1/EPB cells and the 4T1/EPB cell-derived UPs contained various new drug resistance- and CSC-associated proteins and other undefined proteins, which might be processed into multiple antigenic peptides and targeted by TAA-specific TCRs. These features might contribute to the enhanced anti-tumor capacity of 4T1/EPB nanovaccine by broadening and enhancing the specific T cell responses against breast cancer in mice.

### Enhanced Infiltration of CD8^+^ CTLs With Extended TCR Repertoire Diversity and Anti-Tumor Effects of UPs-4T1/EPB Nanovaccine in 4T1/WT and 4T1/EPB Tumor-Bearing Mice

**Figure 2:**
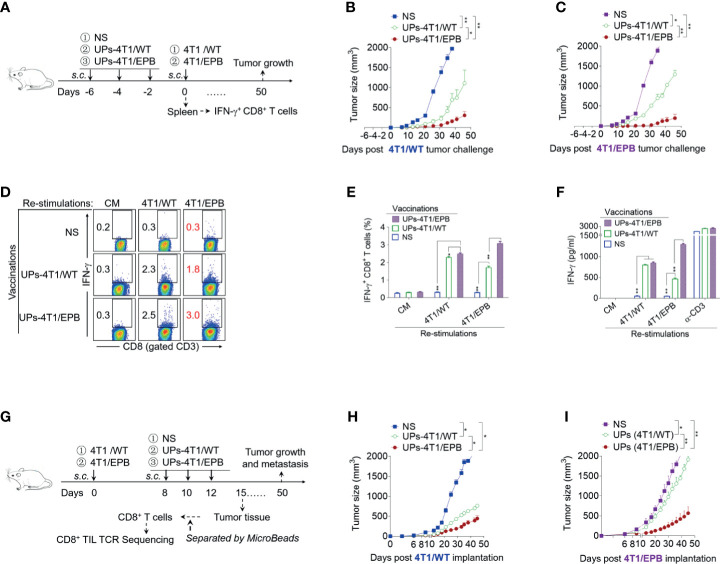
Ubiquitinated proteins (UPs)-4T1/epirubicin (EPB) nanovaccine induced strong anti-tumor effects in 4T1/WT and 4T1/EPB tumor-bearing mice. **(A)** The immunization protocol of UP vaccination for the prophylactic treatment. BALB/c mice received a s.c. injection of NS, UPs-4T1/WT nanovaccine, or UPs-4T1/EPB nanovaccine three times at 2-day intervals. Two experiments were performed concomitantly, one in which the mice (*n* = 7 per group) received tumor challenge with 5 × 10^5^ 4T1/WT cells or 4T1/EPB cells 2 days after the last immunization and one in which splenocytes were harvested after the mice (*n* = 5 per group) were sacrificed for further experiment at 1 week after the last immunization. **(B, C)** Tumor growth was monitored after 4T1/WT **(B)** or 4T1/EPB **(C)** challenge. **(D–F)** The splenocytes collected from **(A)** were then stimulated with inactivated 4T1/WT cells or 4T1/EPB cells for 24 h, and the IFN-γ^+^ CD3^+^ CD8^+^ T cells were examined by flow cytometry **(D, E)**. The supernatants were collected and examined for IFN-γ with an ELISA assay **(F)**. **(G)** Schematic diagram of UP vaccination for the therapeutic treatment. A total of 5 × 10^5^ 4T1/WT cells or 4T1/EPB cells were respectively injected into BALB/c mice on day 0. Tumor-bearing mice received a s.c. injection of NS, UPs-4T1/WT nanovaccine, or UPs-4T1/EPB nanovaccine three times on days 8, 10, and 12 (*n* = 12 per group). Six mice were used for observations of tumor growth and survival until day 50. On day 15, six mice from each group in 4T1/EPB tumor-bearing mice were sacrificed, and the tumor tissues were separated for CD8^+^ TIL isolation. **(H, I)** Tumor growth was monitored in 4T1/WT **(H)** or 4T1/EPB **(I)** tumor-bearing mice. *P*-values were determined by Mann–Whitney *U*-test. The results are representative of three independent experiments, and data were expressed as means ± SEM (*p < 0.05; **p < 0.01; ns, not significant).

We first examined the prophylactic effects of UPs-4T1/EPB nanovaccine on 4T1/WT and 4T1/EPB tumor-bearing mice (**Figure 2A**). The prophylactic anti-tumor growth and metastasis efficacy of UPs-4T1/EPB nanovaccine were significantly higher compared to that of UPs-4T1/WT nanovaccine both in 4T1/WT and 4T1/EPB tumor-bearing models (**Figures 2B, C** and **Supplementary Figures S2A–D**). Splenocytes from mice vaccinated with UPs-4T1/WT or UPs-4T1/EPB nanovaccine were restimulated with inactivated 4T1/WT or 4T1/EPB tumor cells, respectively, to investigate whether UPs-4T1/EPB nanovaccine elicited a stronger tumor-specific immune response. Flow cytometry analysis showed that the percentage of IFN-γ^+^ CD8^+^ T cells was significantly higher in the UPs-4T1/EPB nanovaccine group than in the UPs-4T1/WT nanovaccine group, regardless of which 4T1 cells were used for restimulation (4T1/WT restimulation: 2.49 *vs*. 2.30%; 4T1/EPB restimulation: 3.1 *vs*. 1.7%, all *P <*0.05; **Figures 2D, E**). The ELISA assay demonstrated that the IFN-γ expression was significantly higher in the UPs-4T1/EPB nanovaccine group than in the UPs-4T1/WT nanovaccine group in case of restimulation with 4T1/EPB cells (1,293.4 *vs*. 461.8 pg/ml, *p* < 0.05) (**Figure 2F**). Then, we detected the therapeutic efficacy of UPs-4T1/EPB nanovaccine in 4T1/WT and 4T1/EPB tumor-bearing mice (**Figure 2G**). The UPs-4T1/EPB nanovaccine exhibited more potent therapeutic efficacy in terms of anti-tumor growth and metastasis compared to the UPs-4T1/WT nanovaccine both in 4T1/WT and 4T1/EPB tumor-bearing models (**Figures 2H, I** and **Supplementary Figures S2E–H**). The abovementioned results suggest that the UPs-4T1/EPB vaccine-induced specific T cells can effectively recognize 4T1/EPB cells, resulting in effective prophylactic and therapeutic effects in mice with 4T1/EPB.

**Figure 3:**
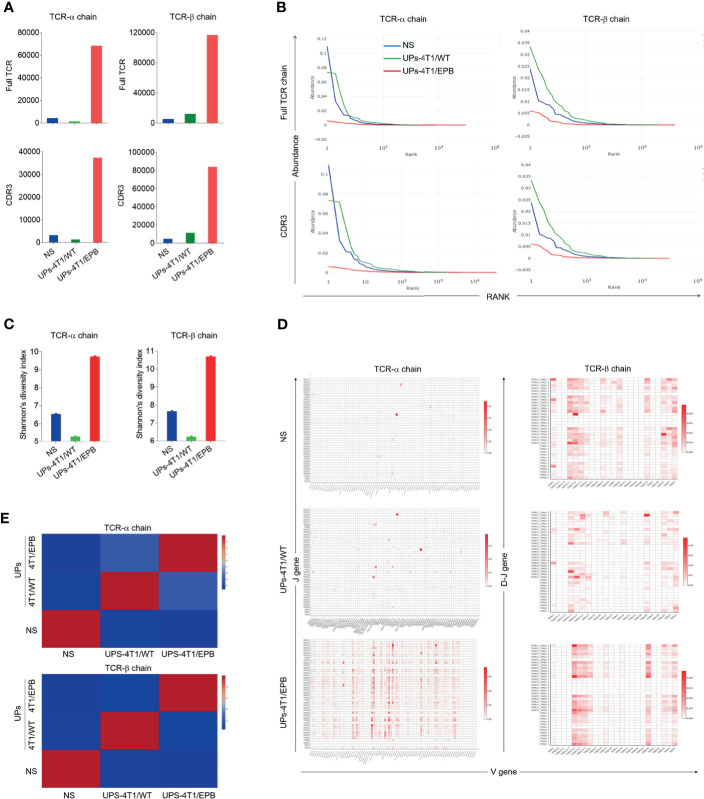
Ubiquitinated proteins (UPs)-4T1/epirubicin (EPB) nanovaccine induced the infiltration of CD8^+^ CTLs with high T cell receptor (TCR) repertoire diversity in 4T1/WT and 4T1/EPB tumor-bearing mice. The TCRs of CD8^+^ TILs from **Figure 2G** were profiled with high-throughput TCR sequencing. **(A)** Number of distinct TCR and CDR 3 clonotypes on TCR αβ chain in different groups. **(B)** The rank abundance curve of TCR and CDR 3 clonotypes on TCR αβ chain in different groups. **(C)** The Shannon diversity index of TCR repertoire on TCR αβ chain in different groups. **(D)** V-J gene usage heat maps for TCR α chain and V-DJ gene usage heat maps for TCR β chain of CD8^+^ TILs in different groups. **(E)** Heat map of similarities in CDR 3 aa usage among different TCR αβ repertoires analyzed by Pearson correlation. NS, normal saline.

The TCR diversity of CD8^+^ TIL is associated with effective anti-tumor effect (34, 35). We therefore harvested 4T1/EPB tumor tissues from unimmunized mice, UPs-4T1/WT-immunized mice, and UPs-4T1/EPB-immunized mice. The CD8^+^ TILs were isolated to detect their TCR sequences, and their TCR diversity was analyze using high-throughput TCR sequencing technology. We first compared the number of amino acid (aa) sequences on TCR α and β chains and the respective CDRs 1–3 clonotypes from CD8^+^ TILs among various groups (**Figure 3A**). The number of aa sequences on the TCR α and β chains (clonotypes) was as follows: 4,292 and 5,394 in unimmunized mice, 1,422 and 12,229 in UPs-4T1/WT immunized mice, and 68,421 and 116,910 in UPs-4T1/EPB immunized mice. The number of aa sequences on CDR 3 of the TCR α and β chains (clonotypes) was as follows: 3,211 and 4,963 in unimmunized mice, 1,329 and 11,321 in UPs-4T1/WT immunized mice, and 37,347 and 84,094 in UPs-4T1/EPB immunized mice. Similar results were obtained on CDR 1 and CDR 2 of TCR α and β chains (**Supplementary Figure S2I**). The abovementioned results indicate a significantly increased number of aa sequences on TCR α and β chains and their respective CDRs 1–3 clonotypes, especially in CDR 3, which is responsible for recognizing antigen peptides, in CD8^+^ TILs from UPs-4T1/EPB immunized mice. Thus, more diverse TCR repertoires were evidenced in CD8^+^ TILs from UPs-4T1/EPB-immunized mice.

Next, we plotted the rank abundance curves of TCR α and β chains and their respective CDRs 1–3 clonotypes. The X-axis represents the clonotype rank. The Y-axis represents the clonotype abundance (relative content) (**Figure 3B** and **Supplementary Figure S2J**). The rank abundance curve, especially the rank abundance curve of TCR α and β chain and CDR 3 α and β clonotype sequence of unimmunized mice, was mainly composed of concentrated clonotype sequences. The curve width, which represents the diversity of clonotype sequences, is narrow, suggesting the limited diversity of TCR in unimmunized mice. In contrast, the curve of UPs-4T1/EPB-immunized mice was much wider than that of unimmunized mice and UPs-4T1/WT-immunized mice, and the curve distribution of UPs-4T1/EPB-immunized mice was more flat, indicating the extended and dispersed TCR repertoires along with the whole distribution range in 4T1/EPB immunized mice. Thus, UPs-4T1/EPB induced a broader spectrum of CD8^+^ TIL TCR repertoires, which could favor the recognition of TAAs, leading to enhanced cytotoxicity. Moreover, the TCR repertoire diversity was also estimated using the Shannon diversity index. The value of the Shannon diversity index on TCR α and β chains was as follows: 6.53 and 7.65 in unimmunized mice, 5.27 and 6.23 in UPs-4T1/WT-immunized mice, and 9.73 and 10.69 in UPs-4T1/EPB-immunized mice (**Figure 3C**). The Shannon diversity index of UPs-4T1/EPB-immunized mice was higher than that of unimmunized mice and UPs-4T1/WT-immunized mice, which reflected a higher TCR repertoire diversity in UPs-4T1/EPB-immunized mice.

Moreover, the heat map of the V-J gene segment usage of TCR α chain and the V-DJ gene segment usage of TCR β chain displayed huge differences in distinct V-J gene pairs and corresponded frequency of CD8^+^ TILs among unimmunized mice, UPs-4T1/WT-immunized mice, and UPs-4T1/EPB immunized mice (**Figure 3D**). The number of distinct V-J pairs was significantly higher in UPs-4T1/EPB-immunized mice than in unimmunized mice and UPs-4T1/WT-immunized mice. Similarly, there were also significant differences on the number of distinct V-DJ pairs, which reflected the diversity of the TCR repertoires and the corresponded frequency of CD8^+^ TILs between unimmunized mice, UPs-4T1/WT-immunized mice, and UPs-4T1/EPB-immunized mice. The abovementioned results collectively suggest that the UPs-4T1/EPB nanovaccine induced a significantly higher diversity of CD8^+^ TILs TCR.

Among all the CDRs of TCR, CDR 3 is critical for the recognition of specific peptide antigens by T cells. We therefore calculated the Pearson correlation coefficient between these three groups based on the distinct CDR 3 aa sequences and their frequency on TCR α and β chains and plotted related heat maps (**Figure 3E**). Both the CDR 3 α and CDR 3 β heat maps displayed the darkest color block between the UPs-4T1/EPB-immunized group and the unimmunized group, referring to the lowest similarity. A significant color difference on heat maps was also demonstrated between the UPs-4T1/EPB and UPs-4T1/WT immunization groups, especially on CDR 3 β heat map.

WebLogo was used to analyze the characteristics of the CDR 3 aa sequences on the TCR α and β chains of CD8^+^ TILs and the top 10 enriched clonotypes from unimmunized mice, UPs-4T1/WT-immunized mice, and UPs-4T1/EPB-immunized mice (**Supplementary Figures S3A–C**). It was shown that the cloned aa sequences on both TCR α and β chains and the top 10 enriched clonotypes differed significantly between the groups, illustrating that the TCR repertoires of CD8^+^ TILs from these three groups are quite different. The dominant TCR clone of CD8^+^ TILs induced by UPs-4T1/EPB may contain specific TCR clones that can recognize 4T1/EPB, which might be linked with the more efficient anti-tumor effects of 4T1/EPB nanovaccine.

Subsequently, we evaluated the *in vivo* safety of UPs-4T1/WT and UPs-4T1/EPB nanovaccines. The histopathological and TEM analyses showed that no pathological change was observed in the main organs, confirming the safety profiles of UPs-4T1/WT and UPs-4T1/EPB nanovaccines (**Supplementary Figures S4A, B**).

### Effects of Combined UPs-4T1/EPB Nanovaccine and STING Agonist Strategy on Tumor Regression and Metastasis in Mice

**Figure 4:**
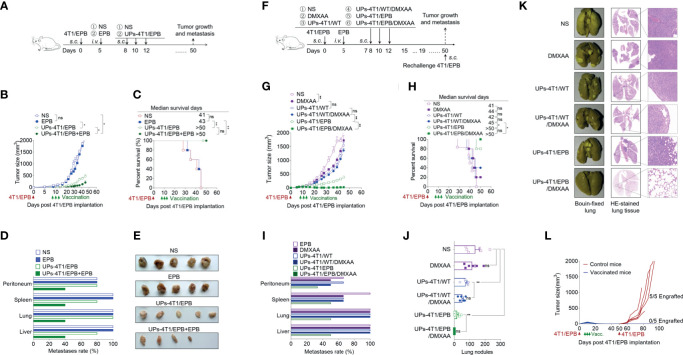
Combination of ubiquitinated proteins (UPs)-4T1/epirubicin (EPB) nanovaccine with chemotherapy and stimulator of interferon genes (STING) agonists led to primary tumor regression and pulmonary metastasis eradication. **(A)** Immunization protocol of UP vaccination combined with chemotherapy. **(B–E)** The tumor growth **(B)** and survival **(C)** of mice (*n* = 5 to 6 per group) were monitored. Metastasis rate **(D)** and tumor photos **(E)** of tumor-bearing mice. **(F)** Immunization protocol of UP vaccination combined with chemotherapy and STING agonists. **(G–L)** The tumor growth **(G)**, survival **(H)**, and metastasis rate **(I)** of tumor-bearing mice (*n* = 6 per group) were monitored. Representative Bouin’s-fixed lungs and microscopic sections of lung tissues with HE stain **(J)**. The pulmonary metastatic nodules of the lung tissue were counted **(K)**. **(L)** The vaccinated mice that rejected the tumor following the combination therapy of UPs-4T1/EPB nanovaccine and 5,6-dimethylxanthenone-4-acetic acid treatment (five of the six treated mice) in **(G)** were rechallenged with 5 × 10^5^ 4T1/EPB tumor cells, and the tumor growth was monitored. *P*-values were determined by Mann–Whitney *U*-test. Kaplan–Meier survival curves were assessed by the log-rank Mantel–Cox test **(C, H)**. The results are representative of three independent experiments, and data were expressed as means ± SEM (*p < 0.05; **p < 0.01; ns, not significant).

Several studies described that the immunogenic cell death primed by EPB chemotherapy could promote tumor antigen presentation followed by tumor-specific T cell responses (36). Accordingly, we tested if combined chemotherapy could enhance the anti-tumor effect of UPs-4T1/EPB nanovaccine or not (**Figure 4A**). As expected, the combinational therapy of UPs-4T1/EPB nanovaccine and EPB possessed enhanced anti-tumor effects than chemotherapy or UPs-4T1/EPB nanovaccine alone, as shown by the retarded tumor growth and reduced metastases in the peritoneum, spleen, lung, and liver from mice challenged with 5 × 10^5^ 4T1/EPB cells (**Figures 4B–E**).

STING agonists have been successfully used as effective vaccine adjuvants and monotherapy agents in several preclinical models (16, 18). DMXAA, a STING agonist, was thus applied in combination with UPs-4T1/EPB nanovaccine in 4T1/EPB tumor-bearing mice (**Figure 4F**). This combination exhibited a dramatic anti-tumor efficacy and resulted in robust and durable tumor regressions in multi-drug-resistant breast tumor in five out of six treated mice (**Figure 4G**). Moreover, all the mice that underwent combination therapy survived to the endpoint (50 days) (**Figure 4H**). Furthermore, the metastases were completely eradicated in mice treated with combination therapy (**Figures 4I–K**). More importantly, none of the cured mice developed new tumors when they were subsequently rechallenged with the same tumor at day 40 after the last immunization, indicating the establishment of tumor-specific immunologic memory (**Figure 4L**). In contrast, anti-tumor efficacy was not enhanced by the combination therapy with DMXAA and UPs-4T1/WT nanovaccine compared to the DMXAA group or UPs-4T1/WT nanovaccine group.

**Figure 5:**
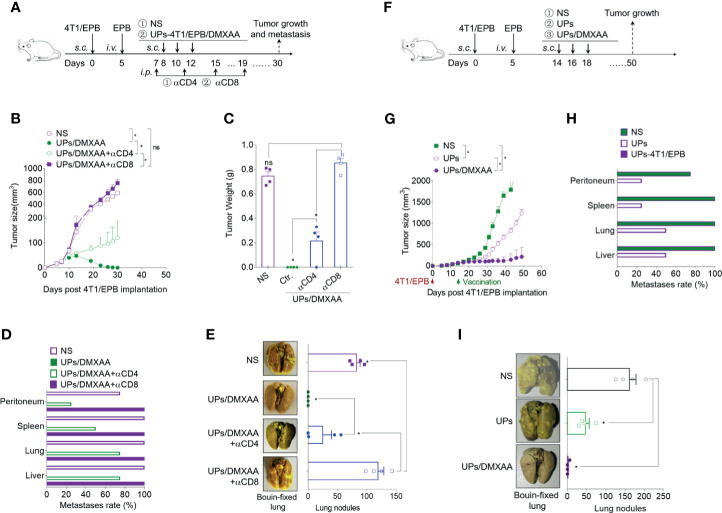
The combination of ubiquitinated proteins (UPs)-4T1/epirubicin (EPB) nanovaccine with chemotherapy and stimulator of interferon genes (STING) agonists exhibited strong anti-tumor effects in larger breast cancer. **(A)** Immunization protocol for UPs-4T1/EPB nanovaccine combined with STING agonists in CD4^+^ T cell-depleted or CD8^+^ T cell-depleted mice (*n* = 4 to 6 per group). **(B–E)** The tumor growth **(B)**, survival **(C)** and metastasis rate **(D)** were monitored. Representative Bouin’s-fixed lungs and the number of lung metastatic nodules in tumor-bearing mice **(E)**. **(F)** Immunization protocol for UPs-4T1/EPB nanovaccine combined with chemotherapy and STING agonists in larger breast cancer (*n* = 4 to 6 per group). **(G–I)** The tumor growth **(G)** and metastasis rate **(H)** were monitored. Representative Bouin’s-fixed lungs and the number of lung metastatic nodules in tumor-bearing mice **(I)**. *P*-values were determined by Mann–Whitney *U*-test. The results are representative of three independent experiments, and data were expressed as means ± SEM (*p < 0.05; ns, not significant).

To confirm if the anti-tumor effects of the UPs-4T1/EPB nanovaccine are dependent on induced CD4^+^ and CD8^+^ T cells, specific antibodies were used to deplete CD4^+^ and CD8^+^ T cells over the course of therapy (**Figure 5A**). The depletion of CD8^+^ T cells in mice led to complete abrogation and the depletion of CD4^+^ T cells in mice led to partial abrogation on the therapeutic efficacy of the combination therapy (**Figures 5B–E**). These data indicate that CD8^+^ T cells and CD4^+^ T cells mediated the anti-tumor effects triggered by the combination treatment with UPs-4T1/EPB nanovaccine and DMXAA.

To evaluate the effectiveness of the UPs-4T1/EPB nanovaccine combined with STING agonist on larger tumors, combinatorial immunotherapy was administered on day 14 after 5 × 10^5^ 4T1/EPB cell implantation (**Figure 5F**). Encouragingly, the combination therapy also showed amazing anti-tumor effects in the mice with larger 4T1/EPB tumor and resulted in complete tumor regression in three out of four mice and in complete elimination of the metastases in all treated mice (**Figures 5G–I**). Taken together, the UPs-4T1/EPB nanovaccine combined with DMXAA option exhibits robust anti-tumor efficacy in this drug-resistant and metastatic murine breast cancer murine model. Moreover, complete tumor regression and metastasis eradication were observed in the majority of mice with larger tumors.

### DMXAA Enhanced the Anti-Tumor Effects of UPs-4T1/EPB Nanovaccine Through Activating CD8α^+^ DC

**Figure 6:**
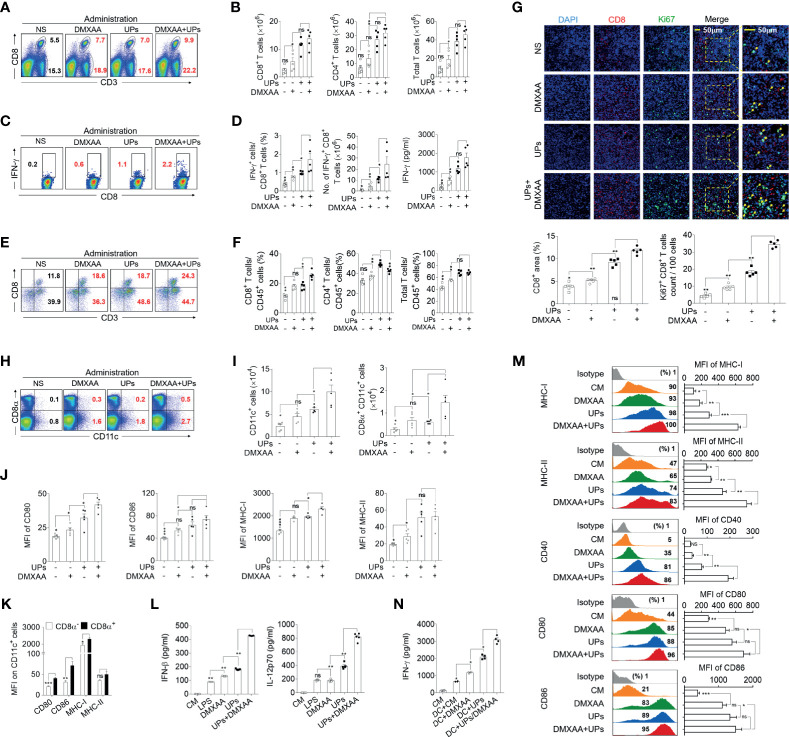
5,6-Dimethylxanthenone-4-acetic acid (DMXAA) enhanced the anti-tumor effect of ubiquitinated proteins (UPs)-4T1/epirubicin (EPB) nanovaccine through the activation, maturation, and lymph node migration of CD8α^+^ dendritic cells (DCs). The mice (*n* = 5 to 6 per group) were challenged with 5 × 10^5^ 4T1/EPB cells s.c. on day 0 and received an i.v. injection of EPB on day 5. The mice received a s.c. vaccination of UPs-4T1/EPB nanovaccine alone or combined with DMXAA three times on days 14, 16, and 18. The mice were euthanized on day 21, and their draining lymph nodes (DLNs), spleens, and tumor tissues were collected for the subsequent experiments. **(A, B)** The representative flow cytometric plots of CD8^+^ T cells, CD4^+^ T cells, and total T cells in spleens **(A)** and the absolute numbers were calculated **(B)**. **(C, D)** The splenocytes were restimulated with inactivated 4T1/EPB cells for 24 h. Representative flow cytometric plots of IFN-γ^+^ CD3^+^ CD8^+^ T cells **(C)**. The percentage of IFN-γ^+^ CD3^+^ CD8^+^ T cells was examined by flow cytometry, and the absolute numbers were calculated. The total IFN-γ level in the cell supernatant was detected by ELISA **(D)**. **(E, F)** Half of each tumor was isolated and processed to a single-cell suspension. Representative flow cytometric plots **(E)**. The percentage **(F)** of total T lymphocytes (CD45^+^ CD3^+^ cells) and T cell subsets (CD45^+^ CD3^+^ CD8^+^ T cells and CD45^+^ CD3^+^ CD8^-^ T cells) was detected using flow cytometry. **(G)** Immunofluorescence microscopy images of DAPI (blue), CD8 (red), Ki67 (green), and merged in the remaining half tumor. The CD8^+^ T cell infiltration area was evaluated using Image J Absolute numbers of Ki67^+^ CD8^+^ T cells were counted in 100 cells per field from five different fields. **(H, I)** Representative flow cytometric plots **(H)** and absolute numbers **(I)** of total DCs and CD8α^+^ dendritic cells (DCs) in DLNs. **(J)** The mean fluorescence intensity (MFI) values of CD80, CD86, and MHC class I and II on CD8α^+^ DCs from different groups. **(K)** The MFI values of CD80, CD86, and MHC class I and II on CD8α ^-^ DCs and CD8α^+^ DCs from UPs-4T1/EPB plus the DMXAA group. **(L, M)** Bone marrow-derived DCs (BMDCs) were cultured with DMXAA, UPs (4T1/EPB), and DMXAA plus UPs (4T1/EPB) after 24 h. IFN-β (top) and IL-12p70 (bottom) were measured in the culture supernatants by ELISA **(L)**. Expression analysis of MHC class I and II, CD40, CD80, and CD86 on BMDCs by flow cytometry **(M)**. **(N)** Splenocytes from UPs-4T1/EPB vaccinated mice were co-cultured with BMDCs from different groups in **(M)** for 12 h, and the IFN-γ secretion in the supernatant was examined by ELISA. Splenocytes alone and splenocytes cocultured with untreated BMDCs served as the controls. *P*-values were determined by Mann–Whitney *U*-test. The results are representative of three independent experiments, and data were expressed as means ± SEM (*p < 0.05; **p < 0.01; ***p < 0.001; ns, not significant); *P <*0.05 was considered significant. All data are presented as mean ± SEM.

Next, we investigated the *in vivo* mechanism mediating the anti-tumor effect of the combination treatment (**Supplementary Figure S5A**). The results showed that both the frequency and the absolute number of CD8^+^ T cells, CD4^+^ T cells, and total T cells in the spleen were significantly higher in mice treated with UPs-4T1/EPB nanovaccine plus DMXAA than in UPs-4T1/EPB-vaccinated mice (**Figures 6A, B** and **Supplementary Figure S5B**). Moreover, intracellular staining results demonstrated that, after stimulation with the relevant inactivated tumor cells *in vitro*, the percentage and absolute number of IFN-γ^+^ CD8^+^ T cells from splenocytes were also significantly higher in mice treated with UPs-4T1/EPB nanovaccine plus DMXAA than in other groups. Consistently, the level of IFN-γ of splenocytes was significantly higher in mice treated with UPs-4T1/EPB nanovaccine or UPs-4T1/EPB nanovaccine combined with DMXAA than in other groups (**Figures 6C, D**). Interestingly, the percentage of CD45^+^ CD3^+^ CD8^+^ T cells (CD8^+^ TILs) in the tumor tissues was significantly higher in mice treated with UPs-4T1/EPB nanovaccine plus DMXAA than in other groups (**Figures 6E, F**). The results of confocal microscopy evidenced much more Ki67^+^ CD8^+^ TILs infiltration in the tumor tissues from mice treated with UPs-4T1/EPB nanovaccine plus DMXAA compared with the other groups (the average numbers of Ki67^+^ cells per 100 CD8^+^ cells are as follows: 34.6 in the group treated with UPs-4T1/EPB nanovaccine plus DMXAA, 18.2 in UPs-4T1/EPB nanovaccine group, and 9.4 in DMXAA treatment group) (**Figure 6G**). Taken together, these results indicate that the combinatorial immunotherapy with UPs-4T1/EPB nanovaccine and DMXAA primed a robust CD8^+^ T cell response in the peripheral immune organ and facilitated CD8^+^ TILs infiltration in 4T1/EPB tumor-bearing mice.

Next, we explored the features of DCs in DLNs of mice treated with UPs-4T1/EPB nanovaccine and mice treated with UPs-4T1/EPB nanovaccine combined with DMXAA. The absolute number and frequency of total DCs and CD8α^+^ DCs were significantly higher in the DLNs from mice treated with the combination therapy compared to the other groups (**Figures 6H, I** and **Supplementary Figure S5C**). The expression levels of activation and antigen-presentation-related markers (CD80, CD86, and MHC class I and II molecules) tended to be higher, and the CD80 and MHC class II molecules were significantly higher on the CD8α^+^ DCs in the DLNs from mice treated with UPs-4T1/EPB nanovaccine or plus DMXAA compared to those in the DLNs from the other groups (**Figure 6J**). The expression of CD80, CD86, and MHC class I and II molecules was also higher on CD8α^+^ DCs compared to those on CD8α^-^ DCs in DLNs from the combination therapy group (**Figure 6K**).

BMDCs were cultured *in vitro* with DMXAA, UPs-4T1/EPB, and UPs-4T1/EPB plus DMXAA, respectively. ELISA assay evidenced the highest IFN-β and IL-12p70 level in the supernatant of the combination therapy group compared to the other groups (**Figure 6L**). Congruent with the *in vivo* data, the highest expression of surface markers (MHC class I and II molecules, CD40, CD80, and CD86) was detected in the combination group with UPs-4T1/EPB and DMXAA (**Figure 6M**). Furthermore, the co-culture UPs-4T1/EPB and DMXAA-loaded BMDCs (APC cells) with splenocytes from UPs-4T1/EPB-vaccinated mice (effector cells) produced the highest level of IFN-γ in the supernatant compared to the co-culture UPs-4T1/EPB or DMXAA alone compared to splenocytes from UPs-4T1/EPB-vaccinated mice (**Figure 6N**). It is important to note that the DC treated with DMXAA can cause IFN-γ responses from splenocytes without an antigen present. This result is in line with previous report in that DMXAA could induce IFN-γ production in unstimulated T cells (37, 38). Taken together, these results suggest that DMXAA, on top of UPs-4T1/EPB nanovaccine, results in more significant activation, maturation, and migration of the UPs-4T1/EPB-loaded DCs to DLN through stimulating the secretion of IFN-β and IL-12, enhancing CD8^+^ T cell responses, and enhancing CD8^+^ TIL infiltration in the tumor tissue, ultimately leading to complete tumor eradication in this model.

Moreover, previous studies collectively demonstrated the anti-tumor efficacy of PD-L1 blockade (39, 40). We thus tested the synergic anti-tumor effects of the combination strategy with UPs-4T1/EPB nanovaccine and PD-L1 blockade in our model (**Supplementary Figure S6A**). As expected, the PD-L1 blockade alone markedly suppressed the growth of subcutaneous 4T1/EPB tumors, but the PD-L1 blockade with anti-PD-L1 antibody did not enhance the anti-tumor efficacy of UPs-4T1/EPB nanovaccine in the UPs-4T1/EPB-vaccinated mice (**Supplementary Figures S6B–E**).

## Discussion

The major findings of the present study are as follows: (1) established a novel EPB-induced multi-drug-resistant cancer stem-like breast cancer cell line (4T1/EPB), (2) enriched proteins were evidenced in UPs from 4T1/EPB cells and prepared the UPs-4T1/EPB nanovaccine, (3) the effective anti-tumor efficacy of this nanovaccine alone and in combination with STING agonist was validated in mice with drug-resistant and metastatic breast cancer, and (4) mechanistically, UPs-4T1/EPB nanovaccine alone and in combination with STING agonist induced the enhanced infiltration of CD8^+^ CTLs in tumor tissue and extended the CD8^+^ TILs TCR repertoire diversity. To the best of the knowledge of the authors, the abovementioned results have not been reported earlier in the literature.

### EPB-Induced Multi-Drug-Resistant Cancer Stem-Like Breast Cancer Cell Line

Previously, EPB-resistant gastric cancer cell lines were reported (41). In this study, we successfully established a new EPB-induced multi-drug-resistant cancer stem-like breast cancer cell line (4T1/EPB). The 4T1/EPB cells exhibited resistance to EPB, cisplatin, Taxol and 5-fluorouracil and significantly upregulated the expression of multiple drug resistance-related genes. Various mechanisms underlying multi-drug resistance have been demonstrated, such as the overexpression of the ATP-binding cassette (ABC) efflux transporters, which extrude structurally and functionally distinct drugs from cancer cells; impaired drug uptake *via* alterations of influx transporters; evasion of apoptosis *via* distinct anti-apoptotic mechanisms; and enhanced DNA damage repair (42). Many literatures also documented that the long-term exposure of tumor cells to one chemotherapeutic drug *in vitro* could induce multi-drug-resistant tumor cell lines (43, 44). In our study, the mRNA and protein expression levels of MDR1 gene were increased in the 4T1/EPB cells (**Figure 1D** and **Supplementary Figure S1B**). MDR1 is responsible for encoding P-glycoprotein, which is one of the ABC efflux transporters that can actively pump chemotherapy drugs out of the tumor cell, thereby reducing intracellular drug accumulation and increasing drug efflux and eventually leading to multi-drug resistance (42, 45). Furthermore, we also found a significantly upregulated expression of BCRP, GST-p, and MMP7 in 4T1/EPB cells. BCRP/ATP-binding cassette subfamily G member 2 (ABCG2) is also an ABC transporter identified as a molecular cause of MDR in diverse cancer cells (42, 46). The GST-π is one of the major detoxification enzymes, which has been reported to concern MDR mechanisms of tumor cells to chemotherapy drugs (47). MMP7 overexpression is related to the enhanced invasive and metastatic capability of MDR tumor cells (48). Thus, upregulated MDR1, BCRP, GST-π, and MMP7 might be the molecular mechanism of induced multiple drug resistance in 4T1/EPB cells.

Moreover, CSCs are related to drug resistance (42). The primary targets for most of the cytotoxic therapies, including chemotherapy, are rapidly dividing, apoptotic sensitive differentiated cells, while CSCs are undifferentiated cells with quiescence reversibility, active anti-apoptotic machineries, and efficient DNA repairing systems (49). Besides this, a hallmark of CSCs is that the cells robustly express drug transporters ABC on the cell surface, with the abovementioned features allowing CSC surveillance and enrichment despite chemotherapy (49, 50). Induction of CSCs through chemotherapy drugs has been applied to numerous solid cancer cell lines, including breast cancer, ovarian cancer, prostate cancer, lung cancer, *etc.* (50). Calcagno et al. (49) have reported that long-term exposure to increasing concentrations of doxorubicin could induce MCF-7-resistant breast cancer cell line MCF-7/ADR with CSC characteristics. Consistent with this report, we defined the CSC features in the newly induced 4T1/EPB cells, as shown by the higher CD44^+^/CD24^−^ cell population and ALDH1 expression (**Figure 1E** and **Supplementary Figure S1C**).

### Enriched Proteins in UPs Derived From 4T1/EPB Cells

In our previous study, we found that UPs derived from tumor cells possess anti-tumor capacities (12, 13). The cellular functions of ubiquitin–proteasome span a wide spectrum, many of which, such as intracellular trafficking, cell cycle, response to oxidative stress, apoptosis, DNA repair, and regulation of enzymatic activity, are directly involved in the processes mediating drug resistance (51). Recent studies have demonstrated that proteasome inhibition enhances the anticancer efficacy of other chemotherapeutic drugs by various mechanisms, such as inhibiting drug efflux transporters, decreasing the expression of anti-apoptotic proteins, activating caspases, and inducing apoptosis; therefore, targeting the ubiquitin–proteasome pathway is thought as a promising strategy to overcome drug resistance (51, 52). Marion L and colleagues (53) compared the protein expression pattern of the MCF-7/ADR cell line to that of the parental MCF-7 cell line using two-dimensional gel electrophoresis followed by mass spectrometry and found that the ubiquitin level was significantly increased in the MCF-7/ADR cell line. Similarly, we found that the number of proteins was about sixfold higher in UPs from 4T1/EPB cells than in UPs from 4T1/WT cells (2,125 *vs*. 362), and drug resistance- and CSC- associated proteins were identified exclusively in 4T1/EPB-derived UPs (**Figure 1I**). The abovementioned findings suggest a highly active state of ubiquitin–proteasome pathway in the drug-resistant cells, which could account for the robust protein expansion that we found in the UPs from 4T1/EPB cells. At the same time, vaccination with 4T1/EPB-derived UPs might be promising due to the significantly enriched UPs (SLiPs).

### Effective Anti-Tumor Efficacy of UPs-4T1/EPB Nanovaccine Alone and in Combination With STING Agonist

Targeting CSCs is thought as the most promising therapeutic strategy, and dozens of clinical trials assessed the effect of drugs and vaccines on the BCSC subpopulation (15). In our study, compared to UPs-4T1/WT nanovaccine, UPs-4T1/EPB nanovaccine displayed a higher anti-tumor efficacy on both 4T1/EPB and 4T1/WT tumors (**Figure 2**). The underlying mechanism could be explained as follows: UPs-4T1/EPB nanovaccine with substantially enriched CSC-associated antigens induced a specific immune response against BCSC and thus resulted in BCSC elimination in both 4T1/EPB and 4T1/WT tumors. The limited clinical benefit of available cancer vaccines might relate to the lack of sufficient TAAs and effective antigen presentation (5, 6). Clinically, triple-negative breast cancer (TNBC) has been reported as the most aggressive molecular subtype with limited treatment methods and a poor prognosis (54). Chemotherapy remains the common treatment for breast cancer; however, the development of drug resistance and the enrichment of CSCs during chemotherapy often lead to the failure of chemotherapy (2). BALB/c-derived murine mammary cell line 4T1, resembling many properties of human TNBC, is widely used as an ideal experimental model for human stage IV metastatic breast cancer (55). Until now there are no EPB-resistant mouse breast cancer cells. In this study, vaccination with UPs-4T1/EPB resulted in satisfactory anti-tumor effects both in the prevention and treatment of 4T1/WT and 4T1/EPB tumor-bearing mice. Enriched SLiPs in UPs-4T1/EPB nanovaccine, which might serve as an efficient TAA source to stimulate specific T cell immune responses and subsequent anti-tumor effects, might significantly contribute the observed effects in both 4T1/EPB and 4T1/WT breast tumor-bearing mice. Accordingly, TILs induced by the UPs-4T1/EPB nanovaccine showed a higher TCR diversity. Some studies have shown that the increase in TCR diversity has been shown to be related with more potent antitumor immunity and tumor clearance (34, 35, 56). In addition, high TCR diversity favors the recognition of the universe of antigenic peptides (57). Hence, the higher diversity of TILs could indicate T cell response covering a broader tumor antigen spectrum. This might be the reason why UPs-4T1/EPB nanovaccine exhibited an effective anti-tumor effect. Therefore, compared to UPs-4T1/WT nanovaccine, UPs-4T1/EPB nanovaccine elicits more diverse and specific T cell immune responses against 4T1/EPB cells.

STING agonists have been successfully used as effective vaccine adjuvants and monotherapy agents in several preclinical models (58, 59). When combined with therapeutic vaccination, a STING agonist can highly enhance the frequency of both peripheral and intra-tumoral antigen-specific effector CD8 T cells along with increased cytotoxicity, resulting in a prolonged control and slower growth of tumors in several tumor models (58, 59). As expected, the application of STING agonist on top of UPs-4T1/EPB nanovaccine amplifies the T cell immune responses elicited by UPs-4T1/EPB nanovaccine. It is important to notice that adaptive therapy resistance could be a major barrier to achieving anti-tumor responses following the direct activation of STING agonist monotherapy (60). Other combinational strategies, such as anti-PD1 and COX2 inhibition, have been reported to enhance the anti-tumor effect of STING agonist in a mouse model of Lewis lung carcinoma (61), indicating that more strategies of adaptive therapy combined with STING agonist need to be tested. Collectively, the combinatorial therapy with chemotherapy, UPs-4T1/EPB nanovaccine, and STING agonist gives rise to amplified T cell responses with higher diversity and specificity against 4T1/EPB cells, resulting in primary tumor regression and metastasis eradication in most mice, even long-term immune protection from tumor recurrence. It is unclear whether the anti-tumor efficacy of the current strategy could be validated in other tumor models, and future studies are warranted to explore this issue.

**Figure 7:**
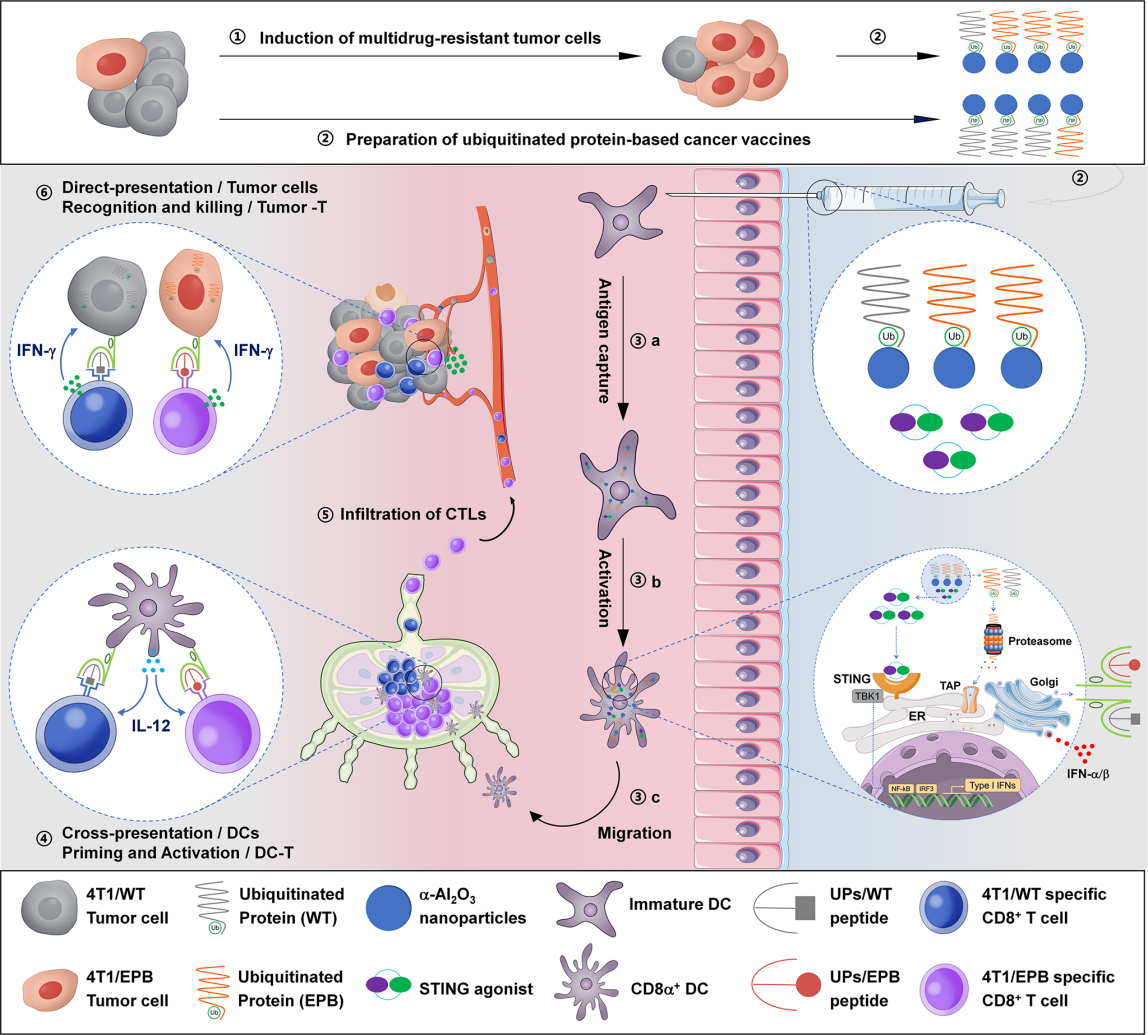
Schematic illustration of the anti-tumor immune responses triggered by ubiquitinated protein-4T1/epirubicin nanovaccine combined with stimulator of interferon gene agonists.

We summarized the immunological mechanism of UPs-4T1/EPB nanovaccine combined with DMXAA in **Figure 7**. The powerful anti-tumor effects of this therapeutic option might be a sum of a series of relevant events as follows: (1) establishment of a multi-drug-resistant cancer stem-like breast cancer cell line (4T1/EPB), (2) preparation of UPs enriched from 4T1/EPB cells as cancer vaccines, (3) DMXAA induced the type I IFN production of DCs, which facilitates antigen processing, activation, maturation, and migration of UP-loaded CD8α^+^ DCs, (4) UP-loaded CD8α^+^ DCs efficiently cross-present UPs to induce 4T1/EPB-specific CD8^+^ T cell responses, (6) effector CD8^+^ T cells migrate and infiltrate to tumor tissue, and (6) CD8^+^ T cells recognized and killed the 4T1/WT and 4T1/EPB tumor cells that directly present peptides from UPs by MHC-I molecules, eventually leading to tumor regression and metastasis eradication in drug-resistant and metastatic murine breast cancer model.

In conclusion, our study demonstrates that vaccination with multi-drug-resistant and CSC-like UPs-4T1/EPB, chemotherapy, and STING agonist strategy is effective for drug-resistant and metastatic breast cancer in mice. Future studies in large animals are warranted to validate the outstanding anti-tumor efficacy of this combination therapy option observed in a murine breast cancer model for future clinical translation. More work is waiting to be done to investigate the therapeutic effects of the tested strategy in breast cancer patients.

## Data Availability Statement

The data presented in the study are deposited in the ProteomeXchange repository, accession number PXD027822. 

## Ethics Statement

The animal study was reviewed and approved by The Institutional Animal Care and Welfare Committee of Southeast University.

## Author Contributions

LW, FH, and NP designed and discussed this research. FH, NP, JZ, YW, ZW, MA, YC, XW, and XS performed the experiments and collected the data. LW and NP provided experimental support. LW, FH, and NP prepared the figures and wrote the manuscript. All authors contributed to the article and approved the submitted version.

## Funding

This work was supported by the National Natural Science Foundation of China (nos. 31670918, 31370895, and 31970849 to LW).

## Conflict of Interest

The authors declare that the research was conducted in the absence of any commercial or financial relationships that could be construed as a potential conflict of interest.

## Publisher’s Note

All claims expressed in this article are solely those of the authors and do not necessarily represent those of their affiliated organizations, or those of the publisher, the editors and the reviewers. Any product that may be evaluated in this article, or claim that may be made by its manufacturer, is not guaranteed or endorsed by the publisher.

## Abbreviations

UPs, ubiquitinated proteins; CSC, cancer stem cell; TCR, T cell receptor; CD8^+^ TILs, tumor-infiltrating CD8^+^ T cells; DLNs, draining lymph nodes; MDR, multi-drug resistance; CTLs, cytotoxic T lymphocytes; DCs, dendritic cells; TAAs, tumor-associated antigens; pAPCs, professional antigen-presenting cells; SLiPs, short-lived proteins; LLiPs, long-life proteins; DRiPs, defective ribosomal products; Dribbles, DRiPs-containing blebs; cGAS, cyclic GMP-AMP synthase; STING, stimulator of interferon genes; ER, endoplasmic reticulum; EPB, epirubicin; IC50, half-maximal inhibitory concentration values; RI, resistance index; s.c., subcutaneous; i.v., intravenous; i.p., intraperitoneal; NS, normal saline; MDR1, multi-drug resistance protein 1; BCRP, breast cancer resistance protein; GST- π, glutathione S transferase- π; MMP7, matrix metallopeptidase 7; CM, culture medium; HE, hematoxylin and eosin; CDR, complementarity-determining region; BMDCs, bone marrow-derived DCs; TEM, transmission electron microscope; BCSCs, breast cancer stem cells; aa, amino acid; ICD, immunogenic cell death; ABC, ATP-binding cassette; P-gp, P-glycoprotein; ABCG2, BCRP/ATP-binding cassette subfamily G member 2; TNBC, triple-negative breast cancer; MFI, mean fluorescence intensity.
