# Supplementary material for: Effects of Combinatorial Ubiquitinated Protein-Based Nanovaccine and STING Agonist in Mice With Drug-Resistant and Metastatic Breast Cancer
Source: Front Immunol. 2021 Sep 13;12:707298. doi: 10.3389/fimmu.2021.707298 (PMC8475273; doi:10.3389/fimmu.2021.707298)
Supplement: Supplementary file 1 [file DataSheet_1.docx]

**Supplementary Materials for**

**Effects of combinatorial UPs-based nanovaccine and STING agonist in mice with drug-resistant and metastatic breast cancer**

**Additional file includes:**

Figures and figure legends. S1 to S6

Supplementary Table. 1 all-peptide Summary of UPs-4T1/WT

Supplementary Table. 2 all-peptide Summary of UPs-4T1/EPB


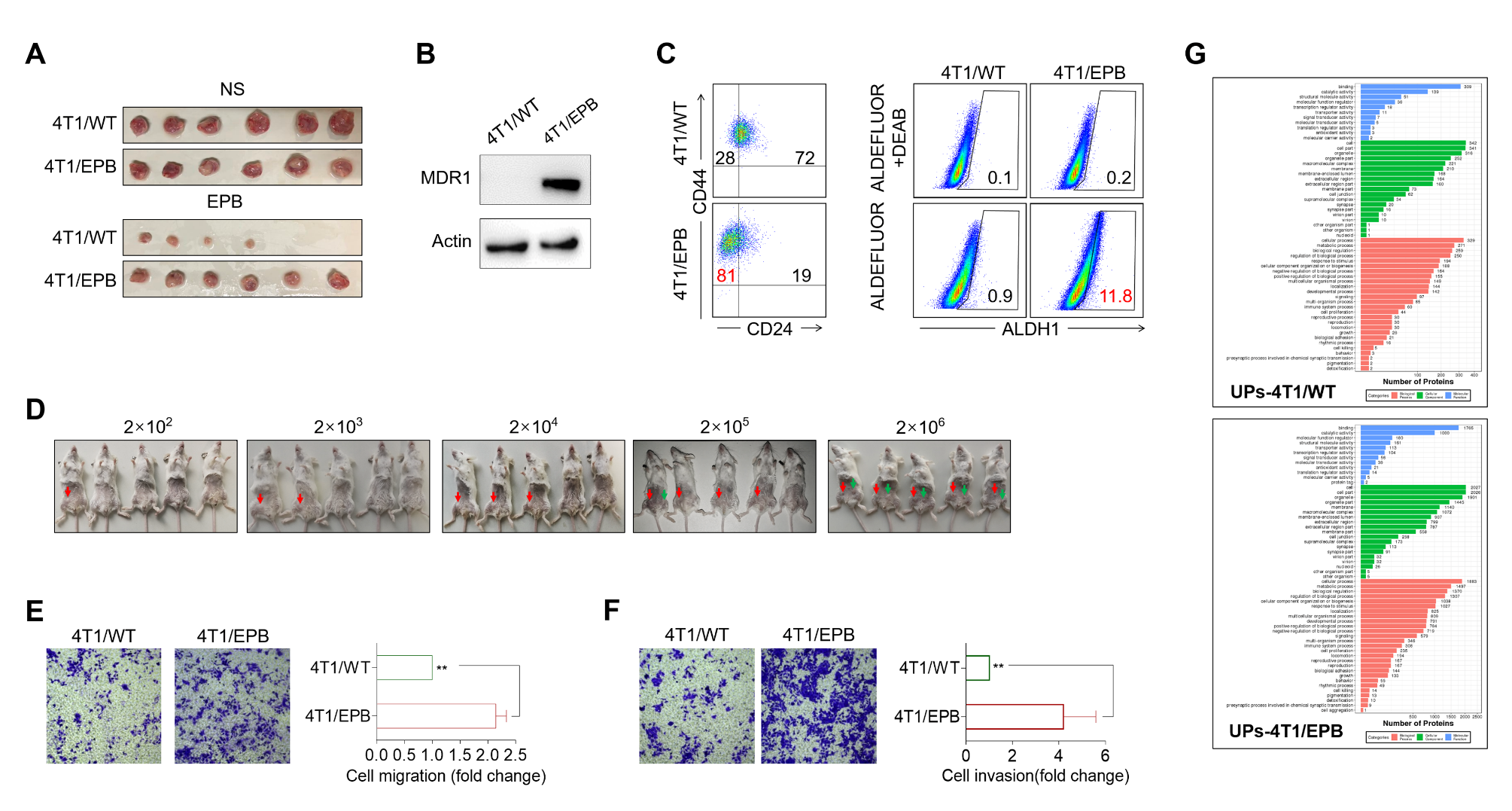


**Figure S1** Multi-drug resistant and BCSCs-like features of the newly established 4T1/EPB cells. **(A)** Tumor photos of each group from Figure 1B. **(B)** Western blot analysis of MDR1 protein expression 4T1/WT and 4T1/EPB cells. **(C)** Representative flow cytometric plots of Figure 1G. **(D)** Representative flow cytometric plots of Figure 1H. **(E, F)** Migration assay and transwell invasion assay were performed in 4T1/WT and 4T1/EPB cells. **(G)** GO functional enrichment analyses of UPs-4T1/WT and UPs-4T1/EPB. P-values were determined by Mann-Whitney U test **(E, F)**. Results are representative of 3 independent experiments and data were expressed as the means ± SEM (*, p < 0.05; **, p < 0.01; ns, not significant).


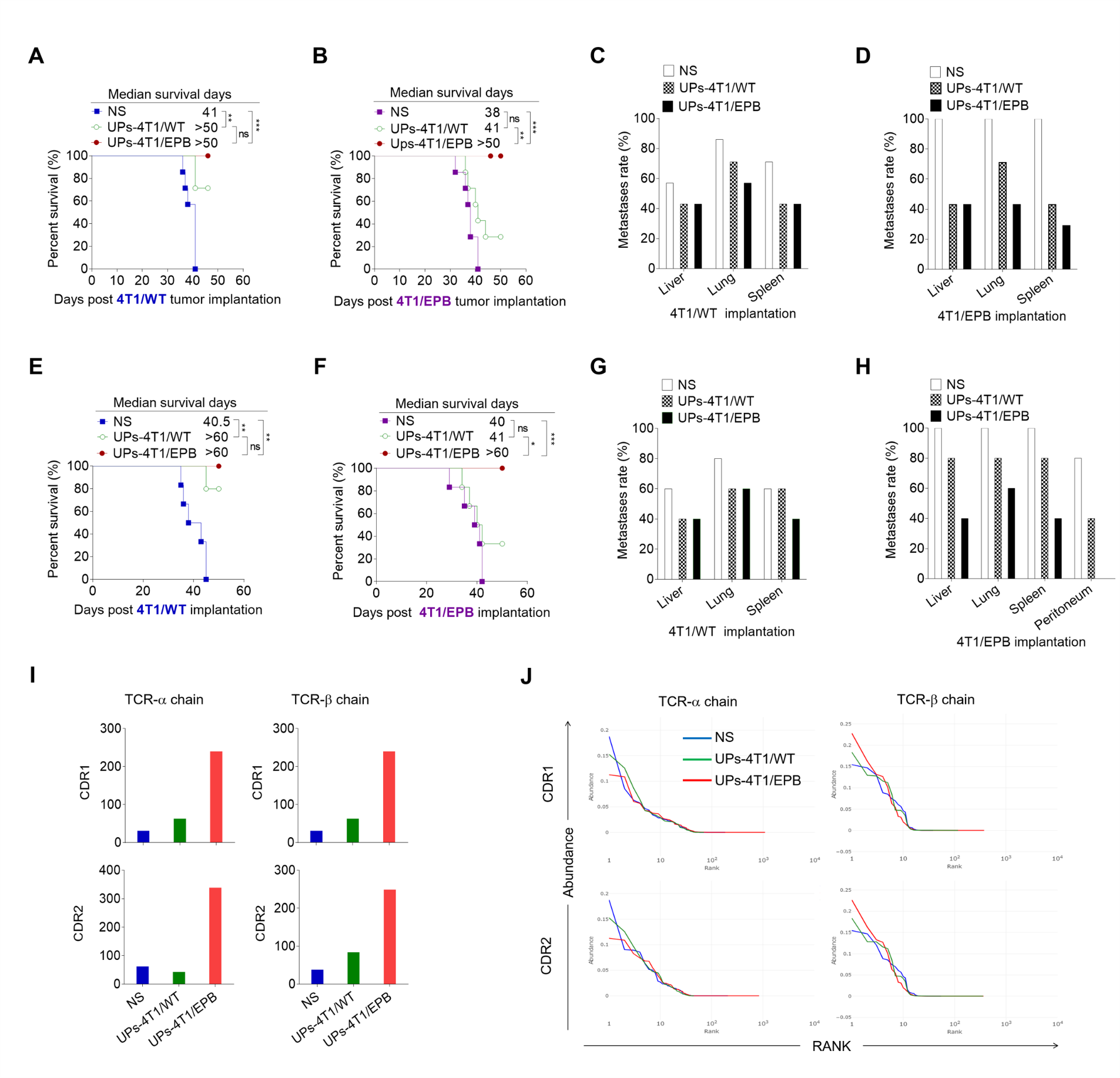


**Figure S2** UPs-4T1/EPB nanovaccine induced strong anti-tumor effects in 4T1/WT and 4T1/EPB tumor-bearing mice. **(A, B)** Survival of mice were monitored after 4T1/WT **(A)** or 4T1/EPB **(B)** challenge from Figure 2A. **(C, D)** Metastasis rate of 4T1/WT **(C)** or 4T1/EPB **(D)** tumor-bearing mice in from Figure 2A. **(E, F)** Survival of 4T1/WT **(E)** or 4T1/EPB **(F)** tumor-bearing mice were monitored from Figure **2G**. **(G, H)** Metastasis rate of 4T1/WT **(G)** or 4T1/EPB (h) tumor-bearing mice in from Figure 2G. **(I)** Numbers of distinct CDR 1 and CDR2 clonotypes of TCR αβ chain in different groups from Figure 3A. **(J)** The rank abundance curve of CDR 1 and CDR2 clonotypes on TCR αβ chain in different groups from Figure 3A. Kaplan-Meier survival curves were assessed by the Log-rank Mantel-Cox test **(A, B, E, and F)**. Results are representative of 3 independent experiments and data were expressed as the means ± SEM (*, p < 0.05; **, p < 0.01; ***, p < 0.001; ns, not significant).


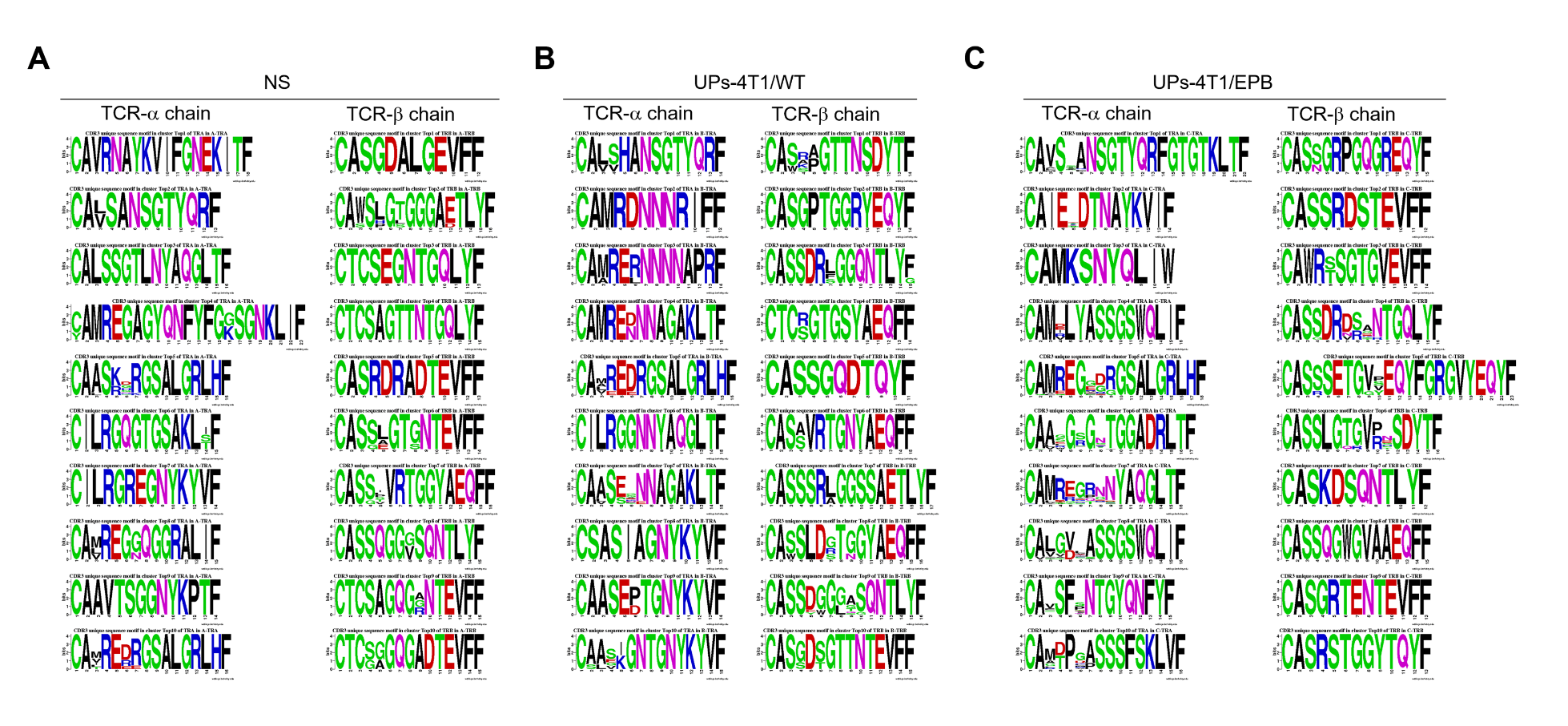


**Figure S3** UPs-4T1/EPB nanovaccine induced the infiltration of CD8^+^ CTLs with high TCR repertoire diversity. **(A-C)** The amino acid distributions of CDR3α (left panel) and CDR3β (right panel) for the TOP 10 CDR3 sequences at the clonal level in NS **(A)**, UPs-4T1/WT vaccination **(B)** and UPs-4T1/WT vaccination **(C)** group from Figure 2G were displayed by WebLogo (http://weblogo.berkeley.edu/logo.cgi).


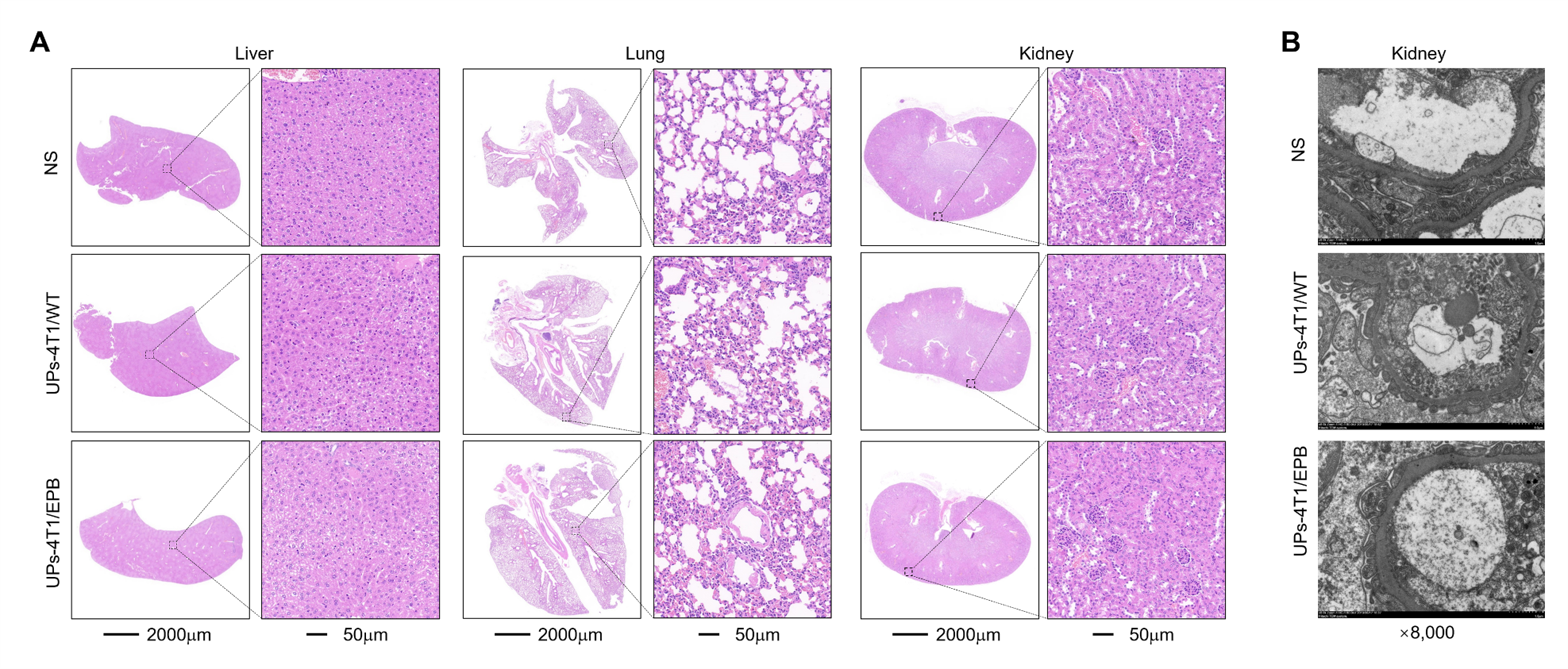


**Figure S4** In-vivo toxicity evaluation of UPs-4T1/EPB nanovaccine on mouse major organs. **(A)** Microscopic sections of liver, lung and kidney tissues with HE stain. **(B)** The ultrastructure of the kidney received different immunization was imaged using TEM.


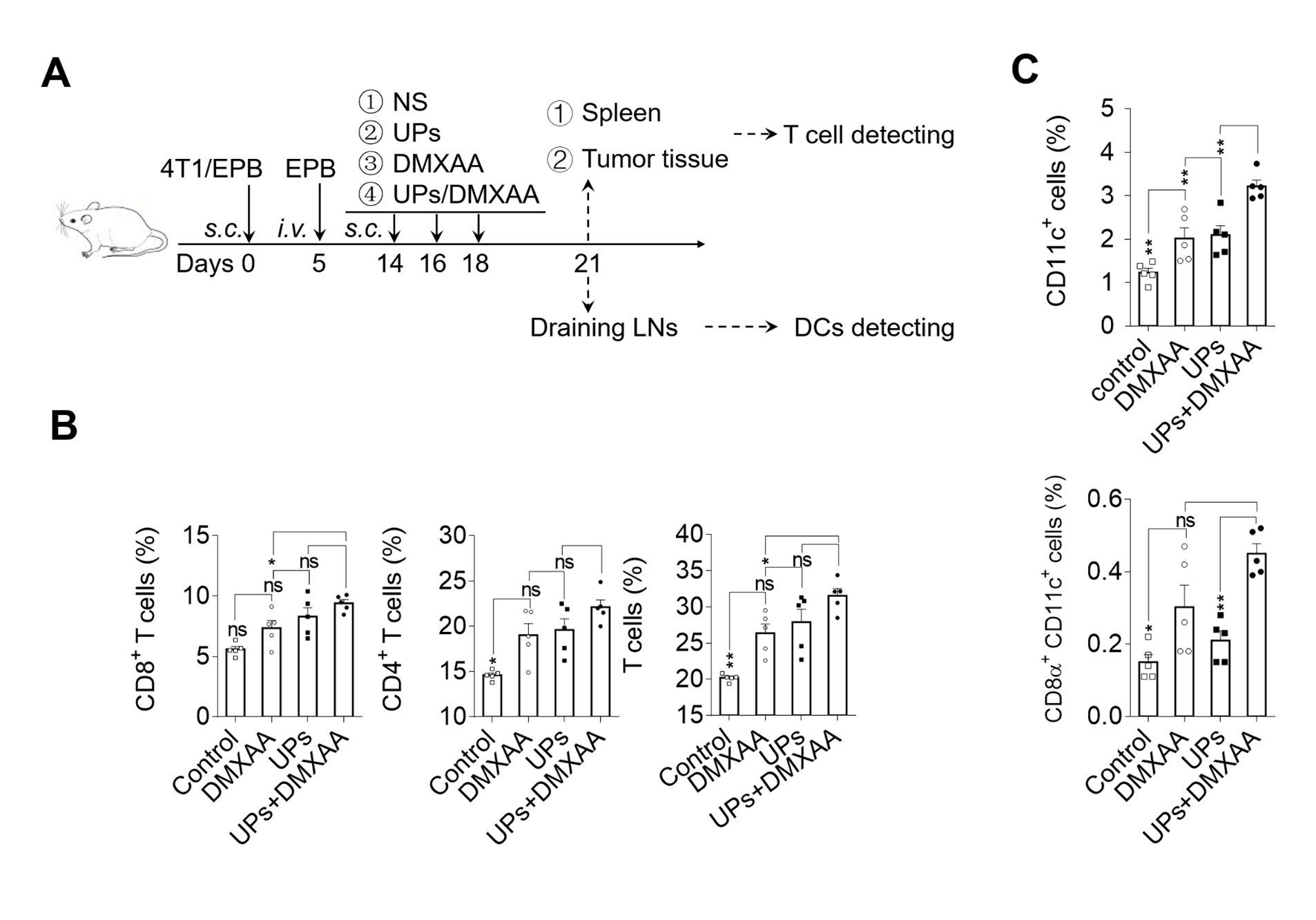


**Figure S5** DMXAA enhanced the anti-tumor effect of UPs-4T1/EPB nanovaccine through the activation, maturation and lymph node-migration of CD8α^+^ DCs. **(A)** Schematic diagram for the in vivo Immune response detection UPs-4T1/EPB nanovaccine combined with DMXAA. **(B)** The percentage of CD8^+^ T cells, CD4^+^ T cells and total T cells in spleens was analyzed by flow cytometry from Figure 6A. **(C)** The percentage of total DCs and CD8α^+^ DCs in draining lymph nodes from Figure 6H. P-values were determined by Kruskal Wallis test **(B and C)**. Results are representative of 3 independent experiments and data were expressed as the means ± SEM (*, p < 0.05; **, p < 0.01; ns, not significant).


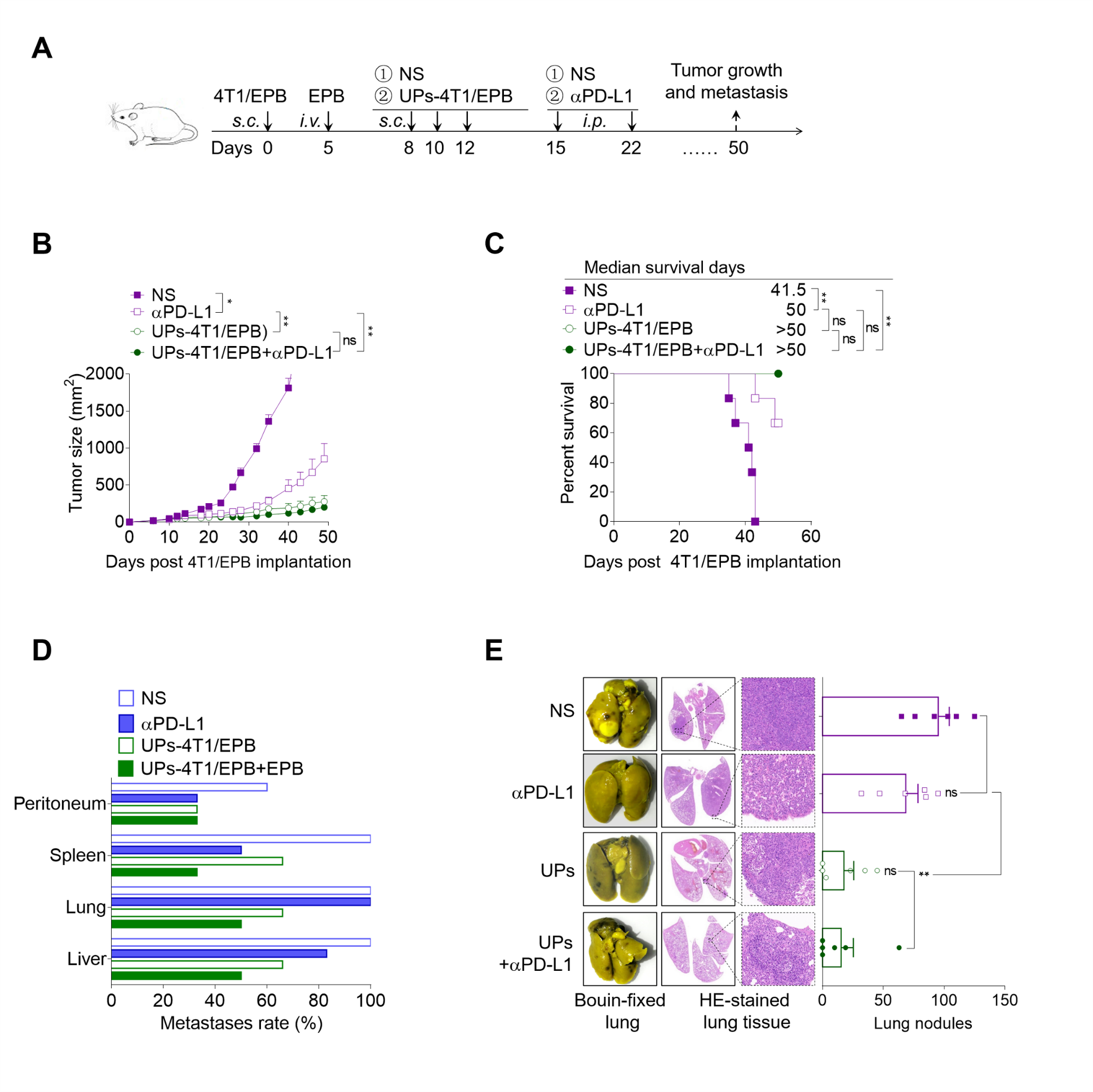


**Figure S6** Anti-tumor effect of UPs-4T1/EPB nanovaccine combined with PD-L1 blockade treatment in 4T1/EPB tumor-bearing mice. **(A)** Immunization protocol for UPs-4T1/EPB nanovaccine combined with chemotherapy and PD-L1 blockade.  **(B-E)** Tumor growth **(B)**, survival **(C)** and metastasis rate **(D)** of tumor-bearing mice (n=6 per group) were monitored. Representative Bouin’s-fixed lungs, microscopic sections of lung tissues with HE stain and the counted number of pulmonary metastatic nodules **(E)**. P-values were determined by Mann-Whitney U test **(B, E)**. Kaplan-Meier survival curves were assessed by the Log-rank Mantel-Cox test **(C)**. Results are representative of 3 independent experiments and data were expressed as the means ± SEM (*, p < 0.05; **, p < 0.01; ns, not significant).
